# PARP1 Activation Controls Stress Granule Assembly after Oxidative Stress and DNA Damage

**DOI:** 10.3390/cells11233932

**Published:** 2022-12-05

**Authors:** Anastasia S. Singatulina, Maria V. Sukhanova, Bénédicte Desforges, Vandana Joshi, David Pastré, Olga I. Lavrik

**Affiliations:** 1 SABNP, INSERM1204, Univ Evry, Université Paris-Saclay, 91025 Evry, France; 2 Institute of Chemical Biology and Fundamental Medicine, Novosibirsk 630090, Russia

**Keywords:** PARP1, stress granules, mRNA translation, oxidative stress, FUS/TLS, TDP-43

## Abstract

DNA damage causes PARP1 activation in the nucleus to set up the machinery responsible for the DNA damage response. Here, we report that, in contrast to cytoplasmic PARPs, the synthesis of poly(ADP-ribose) by PARP1 opposes the formation of cytoplasmic mRNA-rich granules after arsenite exposure by reducing polysome dissociation. However, when mRNA-rich granules are pre-formed, whether in the cytoplasm or nucleus, PARP1 activation positively regulates their assembly, though without additional recruitment of poly(ADP-ribose) in stress granules. In addition, PARP1 promotes the formation of TDP-43- and FUS-rich granules in the cytoplasm, two RNA-binding proteins which form neuronal cytoplasmic inclusions observed in certain neurodegenerative diseases such as amyotrophic lateral sclerosis and frontotemporal lobar degeneration. Together, the results therefore reveal a dual role of PARP1 activation which, on the one hand, prevents the early stage of stress granule assembly and, on the other hand, enables the persistence of cytoplasmic mRNA-rich granules in cells which may be detrimental in aging neurons.

## 1. Introduction

Stress granules (SGs) are dynamic compartments found in the cytoplasm of eukaryotic cells in response to a wide variety of stress, including oxidative stress, hypoxia and viral infections [1]. SGs are considered dynamic and reversible compartments containing non-polysomal mRNAs, 40S ribosomal subunits, translation initiation factors and mRNA-binding proteins (RBPs) [1]. The mRNA and protein composition of SGs varies according to the type of stress at the origin of their formation, probably to coordinate a stress-specific translational response in cells [2,3]. SGs are formed following the dissociation of polysomes, which is generally orchestrated by dedicated kinases after cellular stress that blocks the initiation of translation [3,4]. The assembly of non-polysomal mRNAs in SGs is subsequently regulated by base pairings between mRNA molecules [5] and interactions between self-associated RBPs such as G3BP-1 [6,7,8], which form a bridge between molecules of mRNA. Recently, several reports have indicated that ADP-ribosyl transferases (for example, tankyrase (PARP5a), PARP12, isoforms PARP13 (1 and 2), PARP14 and PARP15) could localize to SGs and play a critical role in the regulation of cytoplasmic SG formation [9,10,11,12]. ADP-ribosyl transferases of the diphtheria toxin-like family (ARTD), also called PARPs, catalyze the transfer of ADP-ribose units from NAD^+^ onto amino acid residues of target proteins, resulting in their (ADP-ribosyl)ation [13]. Some PARPs have the ability to covalently attach a single ADP-ribose to an amino acid residue (protein mono(ADP-ribosyl)ation, MARylation), while others attach a poly(ADP-ribose) (PAR) chain, linear or branched (protein poly(ADP-ribosyl)ation, PARylation). Since SGs are formed in the cytoplasm, most research was focused on cytoplasmic PARPs such has as PARP5a and PARP5b [12]. The action of cytoplasmic PARPs generally positively regulates SG formation and alters their dynamics [9,11,12]. In agreement with this model, the degradation of PAR by cytoplasmic poly(ADP-ribose) glycohydrolase (PARG), namely PARG99 and PARG102, facilitates the dissociation of SGs, thus providing a reversible and controlled mechanism of SG assembly [14]. In addition, many protein components of SGs, such as FUS/TLS (Fused in Sarcoma/Translocated in LipoSarcoma) [15], TDP-43 (TAR DNA-binding protein 43) [12], Y-box binding protein 1 [16], hnRNP A1 (Heterogeneous nuclear ribonucleoproteins A1) [9], can bind PAR or/and can be PARylated by PARPs. Even if the precise role of the post-translational modifications operated by the cytoplasmic or nuclear PARPs remains to be defined, two scenarios have been proposed: (i) PARPs modify RBPs, such as G3BP-1 (Ras-GTPase-activating protein (GAP)-binding protein 1), Ago2 (Argonaute RISC Catalytic Component 2) and hnRNP A1, which then upregulate the formation of SGs [9,11]; (ii) the chains of PAR could serve as a scaffold to recruit self-associated RBPs, such as FUS, TDP-43 and G3BP-1, which promote SG formation [12,17]. Consistently, with the last scenario, purified PAR could promote liquid–liquid phase separation of SG-associated RBPs such as FUS, TAF15 (TATA-Box Binding Protein-Associated Factor 15) and EWS (Ewing Sarcoma protein) [15]. 

Here, we explore the role of the major nuclear PARP, PARP1, in the formation of SGs under oxidative stress induced by arsenite and hydrogen peroxide (H_2_O_2_). Oxidative stress reagents could cause damage to DNA, proteins and lipids and can disrupt biochemical processes and organelle functions vital for cellular survival that can lead to the development of diseases and disorders, including cardiovascular diseases, chronic obstructive pulmonary disease, chronic kidney disease, neurodegenerative diseases and cancer [18,19]. The synthesis of PAR by PARP1 occurs after DNA damage, in particular, in single-strand breaks, which takes place throughout the life of cells, but the occurrence of which increases sharply during oxidative stress [20]. PARP1 is responsible for about 90% of the PAR synthesized in cells in response to genotoxic stress [21] but, surprisingly, little is known about its contribution to SG assembly [11,22]. PAR is covalently attached to acceptor proteins, notably PARP1 itself, but also to other nuclear proteins involved in DNA and RNA metabolism [23,24,25,26]. PAR massively produced during oxidative DNA damage may directly or indirectly participate in the regulation of translation accomplished by the formation of SGs in the cytoplasm [10]. In this study, we considered the putative link between the activation of PARP1 and the formation of mRNA-rich SG in the nucleus and cytoplasm of HeLa cells under oxidative stress induced by H_2_O_2_. In addition, recent reports suggest a critical role of PARP1 in certain neurodegenerative diseases such as sporadic and familial forms of amyotrophic lateral sclerosis (ALS) and frontotemporal degeneration (FTD), since poly(ADP-ribose) is implicated in the accumulation of proteins causative of neurodegenerative disease in the motor neurons [26,27,28,29]. Therefore, particular attention was also paid to the PARP1-dependent formation of mRNA-rich granules containing two RNA-binding proteins, TDP-43 and FUS, since both TDP-43 and FUS translocated from the nucleus to the cytoplasm after H_2_O_2_ treatment [30]. In addition, TDP-43 and FUS are linked to the pathogenesis of neurodegenerative diseases since they form cytoplasmic inclusions in neurons of ALS or FTD patients [31,32].

By analyzing SG assembly in cells, we observed that H_2_O_2_-induced stress prevents the formation of SGs in the cytoplasm in a PARP1-dependent manner. The analysis of mRNA associated with polysomes revealed that H_2_O_2_-induced PARP1 activation limits the dissociation of polysomes, consistent with an inhibition of SG assembly. However, when SGs were pre-formed in the cytoplasm or the translation was terminated, nuclear PARPs do positively regulate SG formation and render them persistent in the cytoplasm. In addition, when TDP-43 or FUS overexpression led to the formation of reversible mRNA-containing granules in the cytoplasm, nuclear PARP activation again promotes their formation. Interestingly, an increased presence of PAR in SGs and TDP-43- or FUS-rich granules was not detected upon H_2_O_2_-induced nuclear PARP activation.

Altogether, our data indicate that PARP1 activation after genotoxic stress may block SG formation if they are not pre-formed but constitutes an aggravating factor in neurodegeneration by preventing the dissociation of pre-formed mRNA-rich granules. In the latter case, nuclear PARPs may indirectly promote the persistence of mRNA-rich protein condensates in the cytoplasm, for instance, via the PARylation of SG-related proteins, phosphorylation events or by buffering mRNA-stabilizing factors such as YB-1 or HuR.

## 2. Materials and Methods

### 2.1. Plasmid and siRNA Transfection 

Fresh culture medium with 5% serum was added to the cells before the transient transfections. HeLa cells were transfected with 1.5 μg/mL of FUS-GFP or TDP-43-GFP encoding plasmids for each well by using the ratio of 1/1000 Lipofectamine 2000 (Thermo Fisher Scientific, Waltham, MA, USA, #11668019) and medium, respectively. The cells were analyzed at 24 h after plasmid DNA transfection. The efficiency of transfection was controlled by immunofluorescence.

For silencing, HeLa cells were then transfected with 20 nM of small interfering RNA duplex FlexiTube GeneSolution for PARP1 (QIAGEN, Hilden, Germany, #GS14) or for PARG (QIAGEN, #GS8505) by using the ratio of 1/1000 Lipofectamine 2000 and medium, respectively. A non-targeting sequence siRNA (AllStars Negative Control siRNA, QIAGEN, #1027281) was used as a negative control. Cells transfected with the siRNA were placed in an incubator for 6 h, then the medium was replaced with fresh medium with 10% FBS, and cells were incubated for 48 h.

### 2.2. Oxidative Stress Conditions and Drug Treatments

For oxidative stress induction, HeLa cells were treated with 300 μM or 1 mM hydrogen peroxide (H_2_O_2_) or 300 μM sodium arsenite for the indicated period of time.

For induction of SGs in nucleus, cells were treated with 4 μM actinomycin D (ActD) (Thermo Fisher Scientific, #11805017) for 1 h before treatment with 300 µM H_2_O_2_. 

For induction of SGs in cytoplasm, cells were treated for 60 min with 300 µM sodium arsenite or 200 µM puromycin (Sigma, St. Louis, MO, USA, #P9620) for 1 h before treatment with 300 µM H_2_O_2_. 

For inhibition of PARP activity, cells were pre-treated with 10 μM olaparib (Apexbio Technology, Houston, TX, USA, #A4154) or 1 μM talazoparib (MedChemexpress, #HY-108413) and diluted in DMSO for 1 h before and during indicated treatments.

To study the disassembly of SGs formed by treatment with sodium arsenite, cells were treated with 300 µM sodium arsenite for 60 min, then the culture medium was replaced with fresh medium containing DMSO (control), 300 µM H_2_O_2_ or 3 µM olaparib or 300 µM H_2_O_2_, as indicated, and then the cells were left alone for one hour for further fixation and analysis.

After incubation with the drugs at indicated time points, the cells were washed with PBS and fixed with 4% paraformaldehyde (PFA) in PBS for 45 min at 37 °C, if another cell fixation method is not indicated.

### 2.3. Immunofluorescence Staining and Image Analysis

For PAR (PAR Affinity Rabbit Polyclonal Antibody, Trevigen, Gaithersburg, MD, USA, #4336-BPC-100) or MAR/PAR (Poly/Mono-ADP Ribose (E6F6A) Rabbit mAb, Cell Signaling, Danvers, MA, USA, #83732) immunofluorescence staining, cells were fixed with 100% ice-cold methanol for 15 min at −20 °C and then washed with PBS and additionally fixed with 4% paraformaldehyde (PFA) in PBS for 20 min at 37 °C. After washing with PBS, coverslips were kept with blocking buffer (50 mM Tris pH 7.5, 100 mM NaCl, BSA 2%, 0.15% Triton X-100) for 40 min at 37 °C in order to permeabilize the cells and reduce nonspecific recognition by antibodies. Blocking buffer was removed and cells were washed and then incubated for 1 h at room temperature with primary antibodies diluted in blocking buffer. The cells were washed 5 times with PBS and incubated for 1 h with secondary antibodies (Goat anti-Rabbit IgG (H + L), Alexa Fluor 594, #11012 or Goat anti-Mouse IgG1, Alexa Fluor 488, #21121, both from Thermo Fisher Scientific) in 50 mM Tris pH 7.5. After final washes with PBS, the cells were stained with 300 nM DAPI to visualize the nuclei and mounted for fluorescence microscopy analysis.

For anti-HA-tag (HA-Tag Antibody, Santa Cruz, CA, USA, #sc-7392) or anti-PARP1 (rabbit polyclonal, Santa Cruz, CA, USA, #sc-7150) immunofluorescence staining, cells were washed with PBS and fixed with 4% paraformaldehyde (PFA) in PBS for 45 min at 37 °C.

The measurements of PAR, MAR/PAR, FUS, TDP-43 and mRNA inside SGs and nuclear granules were defined as the immunofluorescence signal of anti-PAR, anti-PAR/MAR, anti-HA-tag or mRNA (oligo d(T)40 Cy3-conjugated oligonucleotides, Sigma) minus the integrated intensity in the surrounding cytoplasmic (or nuclear) background and were performed using the “CellProfiler” software (version 4.0.7), Broad Institute, Cambridge, MA, USA, https://cellprofiler.org/, 2020 year). SGs, nuclear granules and protein-rich condensates were identified using the “CellProfiler” software as areas close in shape to a sphere or oval and in which the intensity of the detected mRNA exceeded the intensity of the background by 1.2–2 times or more.

### 2.4. RNA Hybridization In Situ

In situ hybridization was performed to image poly(A) mRNA in HeLa cells as follows. Cells were fixed with 4% PFA as explained above. Cells were then incubated with 100% ice-cold methanol for 10 minutes at −20 °C, in ice-cold 70% ethanol for 10 min at −20 °C, and then 1 M Tris-HCl pH 8.0 for 5 min. Poly(T) conjugated to Cy3 (cy3-labelled poly(dT), 40 nucleotides) at 1 μg/μL in the hybridization buffer (0.005% BSA, 1 mg/mL yeast RNA, 10% dextran sulfate, 25% formamide in 2XSSC) was then used to reveal mRNAs. Slides were then placed in a humidity chamber for 1 h at 37 °C with gentle shaking. Following hybridization, cells were washed twice with 4X SSC buffer and once with 2X SSC buffer. After cells were stained with 300 nM DAPI to visualize the nuclei, they were directly mounted for fluorescence microscopy analysis or were proceeded to incubation with primary antibodies (as described above) to obtain immunofluorescence staining of PAR and proteins.

### 2.5. Preparation of Extracts and Polysome Fractionation Analysis

Polysome fractionation analysis was performed as previously described [33]. Briefly, to prepare the cytoplasmic extract, HeLa cells were grown on 15 cm Petri dishes with 70% coverage (approximately 13 million cells per dish) and treated with reagents as indicated and described in the cell culture section. Next, the cells were treated with cycloheximide (Apexbio Technology, #A4154) [33] before cell dissociation from the dish surface according to the standard protocol of trypsin cell culture, after which the cells were centrifuged, lysed and fractionated to obtain cytoplasmic extract by using specific buffer conditions and the procedure fully described in the protocol [33]. After that, the cytoplasmic cell extracts were applied to the sucrose gradient (10–50%) in the buffer conditions according to the protocol [33] and centrifuged at 18,000× *g* rpm in a Beckman SW40Ti swinging-bucket rotor for 11 h at 4 °C. 

Next, the sucrose gradient was fractionated using a “Milichrom” microcolumn liquid chromatograph equipped with a UV detector to monitor the UV adsorption profile at 260 nm in order to obtain a sedimentation curve of the mRNA in the sucrose gradient. Sedimentation curves were drawn (polysome profile) representing the amount (adsorption intensity on 260 nm) of mRNA distributed according to the sedimentation coefficient, which depends on the mass and size of the mRNA and mRNA–polysome complexes. 

The polysome profile for each sample includes fractions without ribosomal material, and fractions with 40S, 60S complexes or 80S (monosome), and low- and high-molecular-weight polysomes. The distribution of mRNA fractions and mRNA–polysome complexes in whole extracts from cells treated with various reagents (300 µM arsenite, 300 µM H_2_O_2_, 200 µM puromycin, 10 µM olaparib.) can be compared between themselves and relative to the control profile obtained in this experiment [33]. For analysis and comparison of the relative amount of polysomes in cell extract samples, the area under the sedimentation curve occupied by the polysome and the 80S monosome fractions in OD_260_ absorbance profile was measured and then normalized to the area under the total absorbance of the sample at 260 nm. The polysomal fraction corresponds to the translated mRNA, and 80S corresponds to the untranslated mRNA fractions and allows us to make a relative assessment of the level of association of polysomes in the cell.

## 3. Results

### 3.1. Activation of PARP1 following Oxidative DNA Damage Prevents Arsenite-Induced SG Formation and Influences Polysome Dissociation

**Figure 1:**
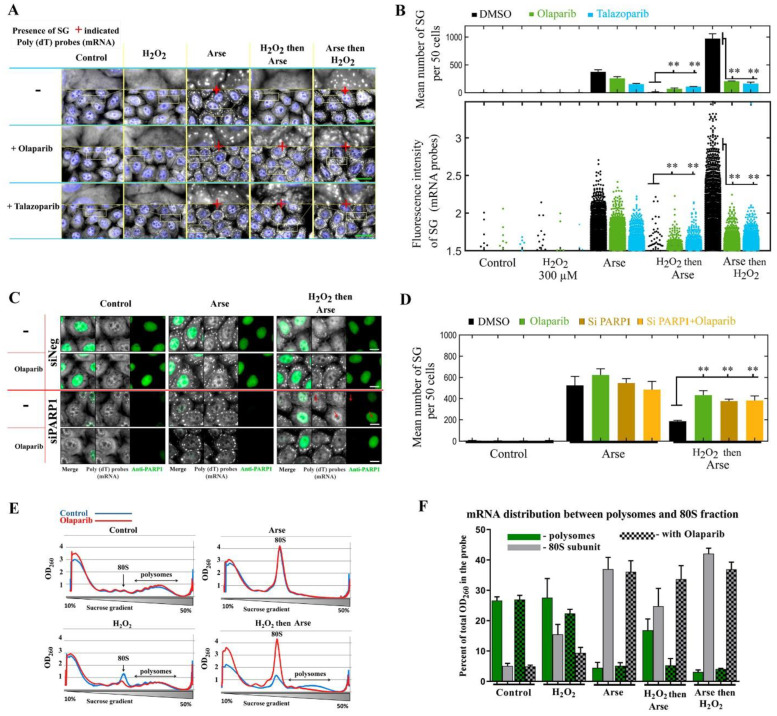
PARP1 activation upon cell exposure to H_2_O_2_ prevents polysome dissociation and thus arsenite-mediated SG assembly. (**A**) HeLa cells were exposed to indicated treatments (300 µM H_2_O_2_ for 30 min; 300 µM arsenite for 60 min). DMSO (control), to inhibit PARP1 10 µM olaparib or 1 µM talazoparib were added 1 h prior to indicated treatment. mRNA was then detected to reveal the presence of SGs in the cytoplasm. Blue: DAPI (nuclei). Grey: mRNA (in situ hybridization with cy3-labelled poly(dT) probe). Scale bar: 10 μm. (**B**) The number of SGs for 50 cells and their fluorescent intensity (in situ hybridization of mRNA) were measured for the indicated conditions. SGs were detected automatically from cell images by using the Cell Profiler software. **, *p* < 0.001; *t*-test. (**C**) HeLa cells were treated with siRNA directed against mRNA encoding PARP1 and then exposed to the same treatments as those indicated in (**A**). Scale bar: 10 μm. (**D**) Mean number of SGs for 50 cells after indicated pretreatment. **, *p* < 0.001; *t* test. Green: anti-PARP1 antibody. Grey: mRNA (in situ hybridization with cy3-labelled poly(dT) probe). (**E**) Polysome profiles obtained from cells treated with arsenite, H_2_O_2_ or H_2_O_2_/arsenite. Cytoplasmic lysates of HeLa cells obtained from indicated conditions were fractionated through sucrose gradients. (**F**) Sucrose gradient (10–50%) profile analysis of mRNA distribution between polysomes and 80S fraction. Fractions corresponding to polysomes and 80S ribosomes are indicated. Treatment is the same as those indicated in (**A**).

Arsenite is a potent inducer of SGs that triggers the rapid phosphorylation of the eukaryotic translation initiation factor 2A (eIF2A), which inhibits translation initiation. The ensuing polysome dissociation resulting from arsenite treatment then leads to the formation of cytoplasmic SGs, in which non-polysomal mRNA is recruited [4,34]. The involvement of cytoplasmic PARPs and PAR in the regulation of SG assembly has been shown for arsenite-treated cells [11]. For this reason, we also considered to investigate the features of SG formation under H_2_O_2_-induced stress conditions, which induce DNA damage and would significantly activate PAR synthesis compared to arsenite [35]. Of note, the assembly of SGs in H_2_O_2_-treated cells has been the focus of many studies [36,37,38,39,40,41]. It turned out that H_2_O_2_ is not a reliable agent to induce SG assembly with either no SG assembly [36,40,42,43] or SG assembly [37,38] being reported. In an extensive study, the appearance of SGs in cells was detected only at a high concentration of H_2_O_2_ (≥1 mM) [36,40]. Therefore, we explored the role of nuclear PAR synthesis in SG assembly in HeLa cells exposed to arsenite, H_2_O_2_, H_2_O_2_ then arsenite or arsenite then H_2_O_2_. A low concentration of H_2_O_2_ (300 µM), which induces PAR synthesis without triggering SG formation, was used for these experiments (Figure 1A,B and Figure S1A,B). Strikingly, when cells were treated with H_2_O_2_ prior to arsenite treatment, the formation of SGs was significantly impaired (Figure 1A,B). In addition, cell treatment with H_2_O_2_ after arsenite exposure led to a strong increase in the number of SGs per cell (Figure 1A,B).

Since H_2_O_2_ not only causes the activation of nuclear PARPs but also an additional stress when combined with arsenite, we wondered whether the inhibition/stimulation of SG assembly in H_2_O_2_/arsenite and arsenite/H_2_O_2_-treated cells was dependent on PARP1 activation. To test this hypothesis, we used olaparib or talazoparib, two PARP inhibitors [44], before inducing cellular stress (Figure 1A,B and Figure S1C). As a control, PARP inhibition moderately reduced SG assembly triggered by arsenite only, which is consistent with the possible participation of cytoplasmic PARPs in SG assembly, whose activities can also be affected by the same inhibitors [11,44]. However, in cells pretreated with PARP inhibitors, H_2_O_2_ and then exposed to arsenite or vice versa, PARP inhibition led to the suppression/stimulation of SG assembly (Figure 1A,B). To further investigate whether nuclear PARP activity inhibits arsenite-mediated SG formation, we decreased PARP1 expression by using siRNA and then analyzed the level of SG formation in cells treated with arsenite or H_2_O_2_/arsenite (Figure 1C,D). PARP1 silencing with siRNA allows the recovery of SG formation in cells treated with H_2_O_2_ prior to arsenite (Figure 1C,D), as observed with PARP inhibitors. Altogether, our findings suggest a negative regulation of SG assembly when H_2_O_2_ treatment causes the activation of PARP1 prior to cell exposure to arsenite.

As H_2_O_2_ treatment does not cause the phosphorylation of eIF2A, in contrast to arsenite [36], we tested the hypothesis that massive eIF2A phosphorylation can be affected by cell pretreatment with puromycin and puromycin/H_2_O_2_ and also in combination with olaparib. We compared the phosphorylation of eIF2A in cells after treatment with arsenite or H_2_O_2_ in the presence or absence of puromycin/olaparib (Supplementary Figure S1D). We noticed a marked increase in eIF2A phosphorylation in cells exposed to arsenite compared to other conditions, but H_2_O_2_, olaparib/arsenite or puromycin/olaparib/H_2_O_2_ has no noticeable influence on the level of eIF2A phosphorylation (Supplementary Figure S1D), which is somewhat in accordance with a previous report [36]. To further decipher the mechanism by which PARP1 activation prevents SG assembly, we explored whether PARP1 could influence the dissociation of polysomes independently of eIF2A phosphorylation. For this purpose, we directly compared the polysome profiles of HeLa cells treated with arsenite, H_2_O_2_ and H_2_O_2_/arsenite in the presence or absence of the PARP1 inhibitor olaparib (Figure 1E,F). As expected, arsenite induced a nearly complete dissociation of polysomes in cells under our experimental condition (Figure 1E). In contrast to arsenite, H_2_O_2_ alone did not significantly affect the overall polysome profile. Interestingly, when cells were pretreated with H_2_O_2_ prior to arsenite, the dissociation of polysomes was noticeably reduced (Figure 1E,F).

This result is consistent with previous studies indicating that H_2_O_2_ treatment may slow down or stall translation elongation, which freezes ribosomes on mRNA [10,45]. Preventing the dissociation of ribosomes from mRNA should suppress SG assembly, as observed after cycloheximide treatment, an inhibitor of translation elongation [4,34]. Importantly, PARP1 inhibition with olaparib neutralized the effect of cell treatment with H_2_O_2_ prior to arsenite, which therefore induces the dissociation of polysomes. Thus, H_2_O_2_-induced PARP1 activation appears to prevent the dissociation of polysomes in arsenite-treated cells independently of changes in the eIF2A phosphorylation status and thereby might prevent the assembly of SGs in the cytoplasm.

### 3.2. Activation of PARP1 Upregulates the Assembly of H_2_O_2_-Induced SGs

**Figure 2:**
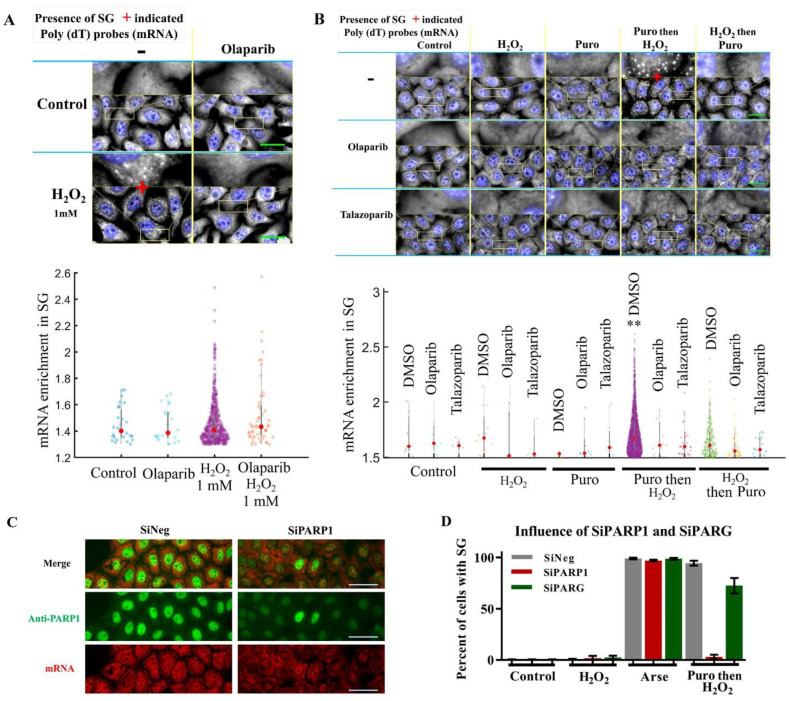
After polysome destabilization by puromycin, PARP1 activation triggers SG assembly. (**A**) HeLa cells were exposed to a high H_2_O_2_ concentration (1 mM for 1 h) to trigger SG assembly. Blue: DAPI (nuclei). Grey: mRNA (in situ hybridization with cy3-labelled poly(dT) probe). Lower panel: the number of SGs detected after cells were exposed to 1 mM H_2_O_2_ decreased sharply when cells were pretreated with olaparib (10 µM). Scale bar: 10 μm. Red dots represent the mean value of the data. (**B**) HeLa cells were exposed to indicated treatments (300 µM H_2_O_2_ for 30 min; 200 µM puromycin 60 min). DMSO (control), 10 µM olaparib and 1 µM talazoparib were added 1 h prior to indicated treatments to inhibit PARP1. mRNA was then detected to reveal the presence of SG sin the cytoplasm. Blue: DAPI (nuclei). Grey: mRNA (in situ hybridization with cy3-labelled poly(dT) probe). Scale bar: 10 μm. Lower panel: mRNA enrichment in detected SGs. **, *p* < 0.001; *t* test. (**C**) HeLa cells were treated with siRNA-directed mRNA encoding PARP1 and then exposed to indicated treatments. Green: anti-PARP1 antibody. Red: mRNA (in situ hybridization with cy3-labelled poly(dT) probe). Scale bar: 10 μm. (**D**) Percent of cells displaying SGs after pretreatments indicated in (**B**).

Since H_2_O_2_-induced PARP1 activation prevents the dissociation of polysomes and SG assembly in arsenite-treated cells (Figure 1), we tested whether PARP1 may also affect the formation of SGs in cells exposed to H_2_O_2_ alone at elevated concentrations. In agreement with another study made using the U2OS cell line [36], we also observed SG formation in HeLa cells only exposed to a high H_2_O_2_ concentration (1 mM) (Figure 2A). 

Remarkably, SGs were not observed in cells treated with 1 mM of H_2_O_2_ in the case of pretreatment with olaparib (Figure 2A). Thus, PARP1 activation promotes SG formation during the stress response to a high concentration of H_2_O_2_ raised to 1 mM. On the one hand, pretreatment with H_2_O_2_ before arsenite impeded SG assembly (Figure 1B). On the other hand, treatment with H_2_O_2_ after arsenite or alone at a high concentration (1 mM) did not prevent SG formation and the dissociation of polysomes (Figure 1A,B and Figure 2A). In order to understand these contrasting effects, further experiments were carried out by stimulating polysome dissociation with puromycin prior to H_2_O_2_ treatment and PARP activation (Figure 2B and Figure S2A). Puromycin induces premature chain termination during translation and promotes ribosome dissociation from mRNA but does not trigger SG assembly by itself [46]. Consistently, puromycin pretreatment before the addition of 300 μM H_2_O_2_ contributes to the formation of SGs (Figure 2B and Figure S2B). The results of the polysome profiling also show a decrease in polysomal mRNA when cells were treated with puromycin before H_2_O_2_ (Supplementary Figure S3). Thus, the pretreatment of HeLa cells with puromycin may counteract the effect of the H_2_O_2_-induced block of polysome dissociation (Supplementary Figure S3B). 

Next, we tested whether PARP1 activation contributes to SG assembly in HeLa cells pretreated with puromycin and then exposed to 300 μM H_2_O_2_. As in the case of cells treated with 1 mM H_2_O_2_, PARP inhibitors prevented SG assembly in puromycin/H_2_O_2_ (300 μM)-treated cells (Figure 2B and Figure S2A). The polysomal profile also showed a slight increase in polysomal fraction and decrease in monosomal fraction (80S) in puromycin/H_2_O_2_-treated cells when PARP activation was inhibited with olaparib, which is consistent with the inhibition of SG assembly (Supplementary Figure S3B). This result indicates that PARP1 activation positively regulates SG assembly after H_2_O_2_ treatment when polysomes are destabilized in the presence of puromycin (Figure 2B, Figures S2 and S3B). To further strengthen this conclusion, we also found that puromycin followed by H_2_O_2_ treatment failed to induce the formation of SGs in HeLa cells treated with PARP1 siRNA (Figure 2C,D). Thus, all results are consistent with an involvement of PARP1 activation in SG assembly caused by both high and low concentrations of H_2_O_2_. 

According to our data, PARP activity can both prevent the formation of SGs by blocking the dissociation of polysomes (Figure 1E,F) and stimulate the formation of SGs if the polysomes are dissociated prior to PARP1 activation (Figure 2 and Figure S2B).

### 3.3. The Presence of PAR in SGs Is not Significantly Affected by PARP1 Activation Whether SGs Were Formed after Arsenite or H_2_O_2_ Treatment

**Figure 3:**
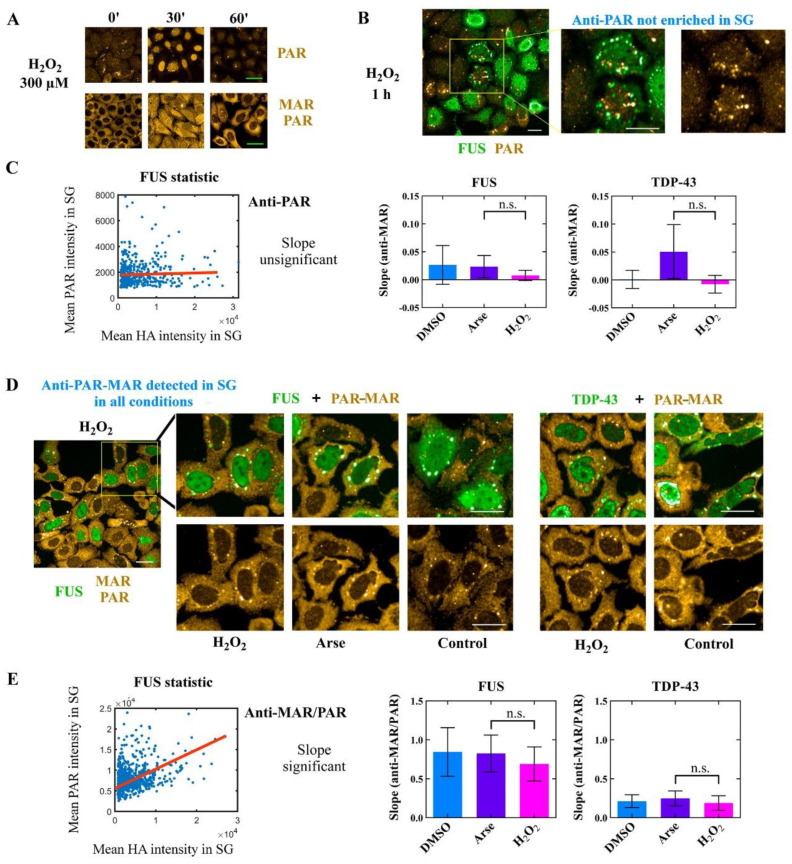
The enrichment of MAR/PAR in SGs is not impacted by oxidative cellular stress (arsenite or H_2_O_2_). (**A**) HeLa cells were exposed to 300 µM H_2_O_2_ for the indicated time. After 60 min exposure to H_2_O_2_, PAR was rapidly digested in the nucleus by PAR-degrading enzymes (PARG and others). Cells were fixed with methanol. Scale bar: 10 μm. (**B**) HA-tagged FUS was overexpressed in HeLa cells to generate SG assembly in the cytoplasm in the absence of additional stress. Then, cells were exposed to DMSO, arsenite or H_2_O_2_ (300 µM for 60 min). Anti-PAR antibody was not detected in SGs. Green: anti-HA antibody. Gold: anti-PAR antibody. Scale bar: 10 μm. Similar results were obtained after 30 min incubation with H_2_O_2_. Confocal images were obtained with an imager Opera Phenix plus. (**C**) Measurement of the slope under indicated conditions (same as (**B**)). Mean enrichment (SG-to-cytoplasmic-intensity ratio) of anti-PAR and anti-HA antibodies was measured in SGs formed after overexpressing either HA-tagged FUS or TDP-43. No influence of the experiment condition on PAR level was detected, n.s.: not significant. (**D**) The same conditions as in (**B**), except the using of anti-MAR/PAR antibody to detect MAR/PAR in the cytoplasm. In this case, MAR/PAR was detected in SGs in all tested conditions. Green: anti-HA antibody. Gold: anti-PAR antibody. Scale bar: 10 μm. (**E**) Measurement of the slope under indicated conditions (same as in (**D**)). A positive slope indicates correlation between the number of HA-tagged proteins recruited to the SGs and MAR/PAR signal, n.s.: not significant.

In cells treated with a high concentration of H_2_O_2_ or puromycin/H_2_O_2_, SG assembly relies on PARP1 activation (Figure 2). Therefore, we wondered whether PAR could be involved in the formation of SGs in this specific condition. As for arsenite [11,22], PAR may play a more prominent role as a scaffold-like molecule in the formation of SGs under H_2_O_2_ treatment. So far, this hypothesis is partially supported by the positive regulation of SG assembly by cytoplasmic PARPs and their downregulation by PARG in the cells treated with arsenite [11]. Although some cytoplasmic PARPs can indeed synthesize long PAR chains [11], to what extent the synthesis of a long polymer would be sufficient to serve as a scaffold for SGs is unknown [10]. PAR has already been detected in arsenite-induced SGs with anti-PAR antibodies, but only when cell fixation was performed after its permeabilization [11]. During the extraction of small soluble molecules, PAR may appear brighter in SGs due to either an enrichment of PAR in SGs or trapping it in SG compartments. To explore whether PARP1 activation after treatment with H_2_O_2_ could contribute to the formation of SG-enriched PAR, we performed a similar experiment but used a methanol fixation rather than PFA, because PFA fixation seems not to be optimal for PAR detection. Indeed, according to previous studies and our experience, PARylation might occur during the cell fixation process with PFA [47]. To perform this experiment, we used five different anti-PAR antibodies, but a transient increase in anti-PAR fluorescence in the nucleus was detected with only two of them within 30 min after exposure to 300 µm H_2_O_2_ (Figure 3A). 

The two validated antibodies were raised against either poly(ADP-ribose) chains (anti-PAR) or against mono- and poly(ADP-ribose) (anti-MAR/PAR), the latter displaying a cytoplasmic localization under the control conditions without H_2_O_2_ treatment (Figure 3A). We failed to detect a significant PAR enrichment in SGs formed in response to either arsenite or puromycin/H_2_O_2_ treatments (Supplementary Figure S4A). Although we did not detect PAR enriched in the SGs of H_2_O_2_-treated cells, both PARP1 and poly(ADP-ribose) glycohydrolase (PARG) activities impact the SG assembly under these stress conditions (Figure 2D and Figure S5). As PARG represents a family of enzymes which is particularly active in digesting PAR chains in both the nucleus and cytoplasm in eukaryotic cells [48], we hypothesize that a rapid degradation of PAR in the nucleus occurs before its putative nucleocytoplasmic shuttling. Thus, PAR may already be hydrolyzed by PARG in the nucleus/cytoplasm for the time required for SG formation (60–90 min upon stress) or short PAR molecules present in the cytoplasm are not recognized by anti-PAR antibodies (Figure 3A).

### 3.4. No PAR Enrichment in TDP-43 and FUS-Rich Condensates in the Cytoplasm

Nuclear PAR produced by PARP1 and protein PARylation could drive the translocation of RBPs from the nucleus to the cytoplasm to promote the formation of SGs. Previously, we demonstrated that H_2_O_2_ treatment affects the nucleocytoplasmic shuttling of SG-associated proteins such as FUS, HuR or TDP-43 [30]. Each of these proteins could contribute to the assembly of SGs, interact with PAR or be PARylated [11,49]. To further probe how PARP1-dependent PAR synthesis may modulate stress granule assembly under H_2_O_2_-induced stress, we generated SGs in HeLa cells by overexpressing HA-tagged TDP-43 or FUS. The presence of these self-adhesive proteins harboring prion-like domains in the cytoplasm is sufficient to trigger the assembly of cytoplasmic granules without oxidative stress [50,51,52,53,54]. Like SGs, the formed granules were shown to be reversible upon cycloheximide treatment and contained mRNAs [4,54]. First, we observed that the level of HA-tagged protein enrichment in the granules remains similar in cells exposed to H_2_O_2_ to activate PARP1 or not, although the number of SGs significantly increased, and its formation was sensitive to PARP1 inhibitors (Supplementary Figure S4B). Next, in the case of the anti-PAR antibody, no correlation in the fluorescence signal was detected between PAR and HA-tagged protein intensity in the SGs of cells overexpressing both FUS and TDP-43 by confocal microscopy (Figure 3B,C). However, a correlation in the fluorescence between MAR/PAR and HA signals in granules was detected with anti-MAR/PAR antibody at least for FUS-rich granules (Figure 3D,E). It is possible that the correlation of PAR intensity with the intensity of HA-labeled FUS in granules, in contrast to TDP-43, may be due to the ability of FUS to be PARylated and(or) to bind PAR with a high affinity and, by this way, attract MAR/PAR to SGs [30]. Compared with arsenite, MAR/PAR enrichment in these granules did not change after the H_2_O_2_-induced activation of PARP1 (Figure 3D,E). Thus, MAR/PAR could be indeed a component of FUS- or TDP-43-rich cytoplasmic granules, although we have no clear evidence that an extensive synthesis of PAR in the nucleus upon PARP1 activation leads to the increased accumulation of PAR in SGs formed with the participation of FUS and TDP-43, which can potentially interact with PAR or be PARylated under oxidative stress.

### 3.5. The Translocation of Nuclear RBPs upon PARP1 Activation May Promote Stress Granule Assembly but FUS Translocation Is Not Enough

To further understand how PARP1 activation and RBPs could promote SG formation, we decided to explore the involvement of endogenous FUS, HuR and TDP-43 in SG formation under H_2_O_2_/puromycin or arsenite stress conditions. It is important to note that the translocation of FUS to the cytoplasm and the retention of HuR in the nucleus was found to depend directly on PARP1 inhibition (Supplementary Figure S6), as previously reported [30]. To test the involvement of endogenous FUS, HuR and TDP-43 in SG formation under H_2_O_2_ treatment, we analyzed the enrichment of these proteins in SGs formed under arsenite or puromycin/H_2_O_2_ treatment (Supplementary Figure S7A). In contrast to HuR, we found that SGs in H_2_O_2_/puromycin-treated cells were enriched in endogenous FUS and TDP-43 compared to arsenite-treated cells (Supplementary Figure S7A). Although TDP-43-rich granules were more abundant in the case of H_2_O_2_-treated cells, the translocation of TDP-43 from the nucleus to the cytoplasm upon treatment with H_2_O_2_ was shown not to be PARP1-dependent [30]; thus, the protein cannot strongly influence the level of H_2_O_2_-induced SG assembly after PARP1 inhibition.

We then considered the possibility that PARP1 could promote SG assembly indirectly through the translocation of FUS, as its translocation has been shown to be dependent on PARP1 [30]. Nevertheless, we found that silencing the expression of FUS by using the CRISPR/Cas9 technology did not prevent the formation of SGs in cells treated with puromycin/H_2_O_2_ (Supplementary Figure S7B). This result is consistent with several genome-wide analyses showing only a few RBPs are critical for SG assembly, FUS not being one of them [55,56]. Alternatively, under H_2_O_2_-induced stress conditions, HuR, a mRNA-stabilizing factor, may act as a negative factor for SG assembly [57], and PAR synthesis in the nucleus may thus favor SG assembly by preventing the translocation of HuR in the cytoplasm (Supplementary Figure S6). 

In summary, SGs formed in the cytoplasm after H_2_O_2_ treatment are enriched in FUS and TDP-43 compared to those formed in the presence of arsenite (Supplementary Figure S7A). Therefore, H_2_O_2_-induced stress influences the composition of SGs. The detection of granules enriched with FUS suggests that this protein could act downstream of PARP1 activation in the process of SG formation, because FUS translocation is strongly dependent on PARP1 activation [30].

### 3.6. PARP1 Activation Promotes mRNA-rich Granule Assembly in the Nucleus without any Mixing with PAR

**Figure 4:**
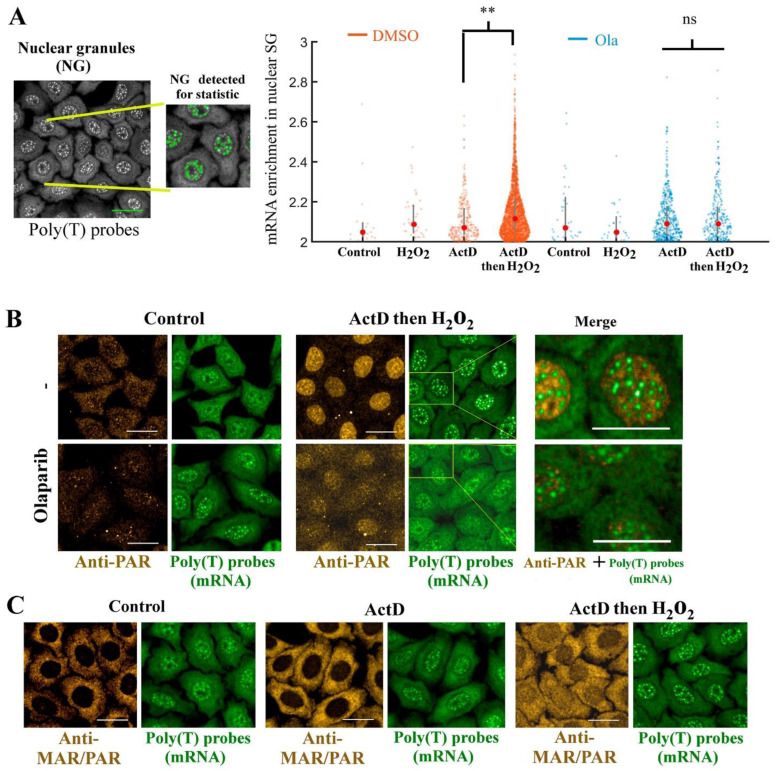
PAR is not detected in mRNA-rich nuclear granules even after PARP1 activation. (**A**) **Left panel**: Automatic detection scheme based on the Harmony software (imager, Opera Phenix plus). **Right panel**: mRNA enrichment in nuclear granules detected under indicated conditions. Scale bar: 10 μm. Red dots represent the mean value of the data, ns: not significant. (**B**) The presence of nuclear mRNA-rich condenses in the nucleus was detected in HeLa cells for indicated conditions (300 µM H_2_O_2_ for 30 min; 4 µM actinomycin D for 1 h prior and during indicated treatment; 10 µM olaparib for 1 h prior to and during indicated treatments). The mRNA enrichment (in situ hybridization with cy3-labelled poly(dT) probes) in the nuclear granules relative to the nucleoplasm was measured, as well as the number of nuclear granules (in quadruplicate). A significant increase in both SG number and fluorescence intensity was detected after combined ActD and H_2_O_2_ treatment; this phenotype was antagonized by olaparib. **, *p* < 0.01; *t* test. Green: mRNA (poly(T) probes). Gold: anti-PAR antibody. Scale bar: 10 μm. (**C**) Representative images of HeLa cells after indicated treatments (those indicated in (**B**)). Note the absence of co-localization between mRNA in nuclear SGs and anti-PAR antibody or anti-MAR/PAR antibody, despite the presence of PAR and MAR/PAR in the nucleus after PARP1 activation by H_2_O_2_ for 30 min. Gold: anti-PAR/MAR antibody. Green: mRNA (in situ hybridization with cy3-labelled poly(dT) probe. Scale bar: 10 μm.

As we did not evidence any enrichment of PAR in cytoplasmic SGs after PARP-1 activation, we then devised to explore whether PAR can be recruited in nuclear SGs, where anti-PAR and anti-PAR/MAR antibodies clearly reveal an increase in PAR levels 30 min after H_2_O_2_ treatment (Figure 3A). In the nucleus, mRNAs are synthetized in the nucleus by RNA polymerase II and then processed by splicing factors [58]. We therefore perturbed these tightly regulated machineries by using actinomycin D (ActD), a general inhibitor of transcription and potent inhibitor of SG formation in the cytoplasm [4,59]. In cells pretreated with ActD, we evidenced the presence of small granule-like structures containing mRNA in the nucleus (Figure 4A). 

We then analyzed the putative recruitment of PAR inside the nuclear mRNA-rich granules observed after ActD or ActD/H_2_O_2_ treatment, when mRNA synthesis was stopped and PARP1 was activated (Figure 4B,C). As observed in the cytoplasm (Figure 3), we found no evidence for an enrichment of PAR in mRNA-rich nuclear granules. We thus did not gather any data to support the presence of abundant long PAR chains in mRNA-rich granules. However, importantly, the nuclear mRNA-rich granules were significantly more numerous and gathered more mRNA inside ActD/H_2_O_2_-treated cells than in ActD-treated cells (Figure 4B,C). Under the same conditions, PARP inhibition by olaparib led to a significant downregulation of mRNA-rich nuclear granules in HeLa cells treated with ActD/H_2_O_2_ (Figure 4A,B), suggesting a common mechanism by which PARP1 activation could positively regulate SG assembly in H_2_O_2_-treated cells, whether in the cytoplasm or the nucleus (Figure 2 and Figure 4). 

Therefore, the activation of PARP1 by DNA damage-induced oxidative stress positively regulates the formation of mRNA-rich granules in the nucleus and cytoplasm of HeLa cells, but without any additional enrichment of PAR in these granules.

### 3.7. PARP1 Activation upon H_2_O_2_ Exposure Negatively Regulates SG Disassembly and Positively Regulates Preexisting TDP-43 and FUS-Rich Granules 

**Figure 5:**
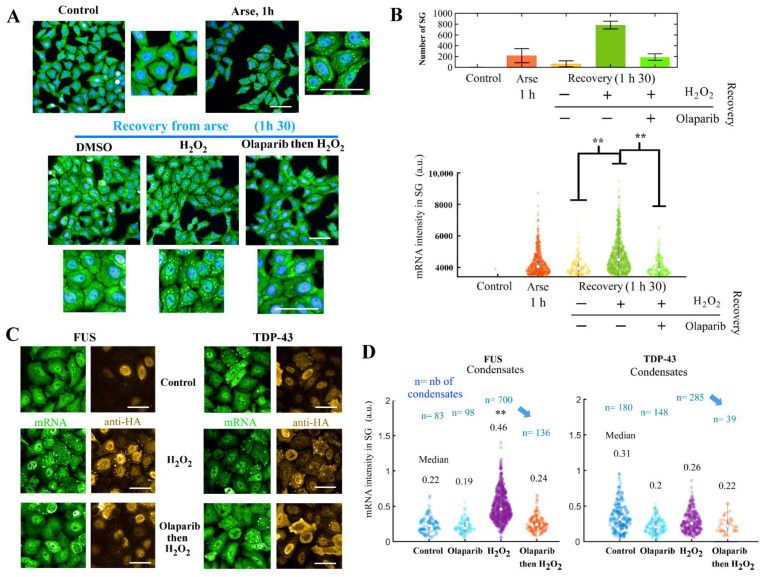
Pre-formed SGs become persistent after PARP1 activation. (**A**) Representative images of HeLa cells exposed to the indicated treatments (300 µM arsenite; 300 µM H_2_O_2_; 3 µM olaparib, 1 h prior to and during indicated treatment). Green: mRNA (in situ hybridization with cy3-labelled poly(dT) probes). Blue: DAPI. Scale bar: 10 μm. During the indicated recovery period, cells were washed out with culture medium with or without olaparib when indicated. (**B**) Automatic detection of SGs reveals an inhibition of SG disassembly during stress recovery when cells were exposed to H_2_O_2_ during the recovery period. mRNA detection: in situ hybridization with poly(dT) probes. **, *p* < 0.01, *t* test. White dots represent the mean value of the data. (**C**) Representative images of HeLa cells overexpressing HA-tagged TDP-43 or FUS exposed to the indicated treatments (300 µM H_2_O_2_ for 1 h; 10 µM olaparib, 1 h prior to and during indicated treatment). Green: mRNA (in situ hybridization with cy3-labelled poly(dT) probes). Gold: anti-HA antibody. Scale bar: 10 μm. (**D**) Analysis of the mRNA intensity in FUS- or TDP-43-rich SGs. The number of condensates (n) in the presence of H_2_O_2_ or other conditions for cells expressing HA-tagged FUS or TDP-43. **, *p* < 0.01, *t*-test. White dots represent the mean value of the data.

As PARP1 positively regulates SG assembly in arsenite/H_2_O_2_- or H_2_O_2_-treated cells when puromycin prematurely disengages ribosomes from polysomes (Figure 1B and Figure 2B), we then considered whether H_2_O_2_ may regulate the dissociation of pre-formed SGs. To this end, we pre-formed SGs in cells treated with arsenite. After that, arsenite was washed out and cells were left to recover with fresh culture medium. SG disassembly was easily captured as the number of cells harboring SGs decreases after 90 min, in agreement with the reversible nature of SGs [60]. However, when cells were treated with 300 µM H_2_O_2_ during the recovery period, the disassembly of SGs was impaired (Figure 5A,B).

In addition, we also noticed that the number of SGs and the mean fluorescence intensity of mRNA is significantly higher in control cells than in cells pretreated with olaparib to prevent PARP1 activation, which may indicate a change in their structure and composition during cell recovery with the H_2_O_2_ treatment (Figure 5B). 

The inhibition of PARP1 activation by olaparib reversed this pattern as fewer SGs and less mRNA were detected inside them (Figure 5A,B). Thus, PARP1 activity prevents the disassembly of pre-existing SGs formed under arsenite treatment, influencing the enrichment of mRNA in SGs. 

We then further explored the hypothesis of the persistence of SGs after PARP1 activation but, this time, in the absence of arsenite pretreatment, which may be the source of biases. Therefore, we again used HeLa cells overexpressing HA-target TDP-43 or FUS, which induces the formation of cytoplasmic granules without stress conditions (Figure 3 and Figure 5C). Although the level of HA-tagged proteins in the condensates remained similar in all conditions (Supplementary Figure S4B), we observed an enrichment of mRNAs in TDP-43 or FUS-rich granules and also an increase in the number of granules after H_2_O_2_ treatment (Figure 5D). Importantly, the increase in mRNA-rich granule assembly in TDP-43 and FUS-overexpressing cells was PARP1-dependent, since these cells pretreated with olaparib displayed a reduced number of SGs even after H_2_O_2_ treatment (Figure 5C,D). 

Therefore, in pre-formed SGs, PARP1 activation delayed SG disassembly upon recovery from arsenite stress (Figure 5A) and upregulated the assembly of cytoplasmic TDP-43 and FUS-positive granules and their enrichment of RNA (Figure 5C).

## 4. Discussion

At present, both PARylated proteins and protein-free PAR (or mono(ADP-ribose)) formed during the action of PARG appears to be involved in the mRNA metabolism-related processes during stress, one of which is SG formation [58]. The involvement of PAR in the regulation of SG assembly in the cytoplasm is a subject of intensive research, which is focused mainly on the PARP, PARG99 and PARG102 isoforms present in the cytoplasm [9,10,11,12]. Nonetheless, the direct role of PARP1 in SG formation has not been fully elucidated. Genotoxic stress causes a strong increase in the cellular PAR level, and this is mainly due to the activation of nuclear PARP1, which is responsible for the synthesis of most PAR in the cell (up to 80–90%) upon DNA damage [21]. At the same time, PARGs, the major enzymes that hydrolyze PAR, are highly active in cells [61]. The half-life of the major population of PAR may be close to one minute, but we cannot exclude that a small fraction of the polymer may have a cell half-life of several hours and be implicated in the cellular adaptation to stress conditions [21,62]. 

**Figure 6:**
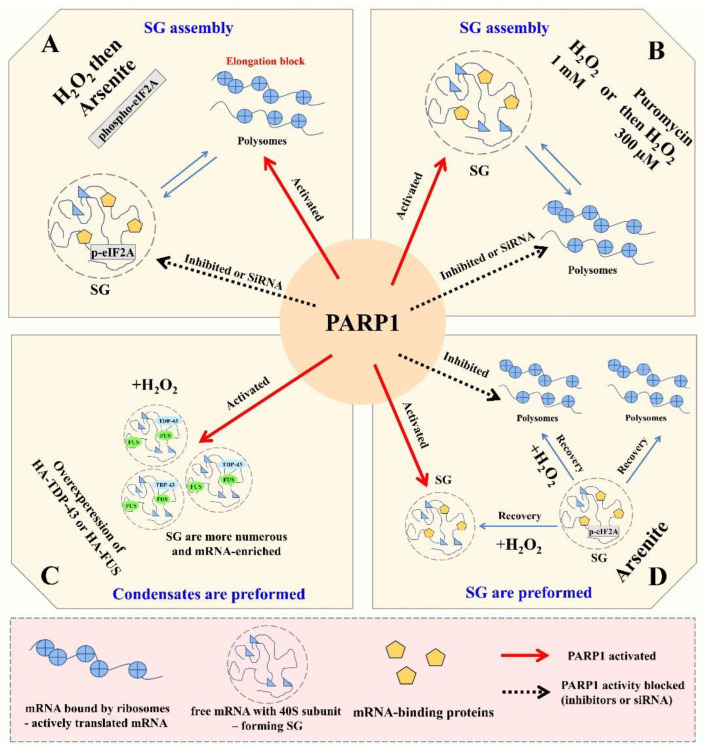
PARP1 could regulate different stages of mRNA-rich granules’ metabolism in cytoplasm. (**A**) PARP1 activation prevents polysomal disassembly induced by arsenite treatment and blocks formation of SGs. (**B**) PARP1 activation stimulates formation of SGs at a high concentration of H_2_O_2_ or if polysomes are destabilized with puromycin. (**C**) PARP1 activation enhances the formation of TDP-43- and FUS-rich SGs under H_2_O_2_-induced stress conditions. (**D**) PARP1 activation upon H_2_O_2_ exposure prevents disassembly of arsenite-induced SGs.

In this context, we investigated the relationship between PARP1 and the formation of mRNA-rich granules in the nucleus and cytoplasm of HeLa cells under different stress conditions induced by H_2_O_2_ treatment alone or in combination with arsenite, transcription inhibitor ActD or translation inhibitor puromycin. Our results indicate a previously unsuspected link between the activation of PARP1 and the efficiency of cytoplasmic mRNA-rich granule assembly under H_2_O_2_-induced stress and suggest a direct relationship between PARP1 activation and translation regulation in mammalian cells (Figure 6). Moreover, we found an association of PARP1 activity with the formation and genesis/maturation of FUS-rich cytoplasmic inclusions, which are found in neurons of patients affected by certain major neurodegenerative diseases, including ALS [32,63,64,65,66]. 

### 4.1. PARP1 Activation Prevents the Assembly of Arsenite-Induced SGs by Preventing Polysome Dissociation

When cells have no pre-formed SGs in the cytoplasm, surprisingly, oxidative stress generated by H_2_O_2_ very efficiently prevents SG assembly, even if arsenite is added afterward, and this effect depends on PARP1 activity (Figure 6A). We found that H_2_O_2_ treatment prevents polysome dissociation, and PARP1 inhibitors in turn restore polysome dissociation in H_2_O_2_/arsenite-treated cells (Figure 1A,B). Thus, this effect of H_2_O_2_ on the stability of polysomes is PARP1 activity-dependent but eIF2A phosphorylation-independent (Supplementary Figure S1D). Therefore, PARP1 activation appears to induce an elongation block, preventing polysome dissociation. A translation elongation block has already been reported after H_2_O_2_ treatment in different organisms, notably via the phosphorylation of elongation factors [45], but not associated directly to nuclear PARP1 activity. Future works may highlight whether PARP1 activation controls the phosphorylation or PARylation status of elongation factors in cells or whether other cellular mechanisms come into play.

### 4.2. PARP1 Activation Upregulates the Assembly of H_2_O_2_-Induced SGs 

The mechanism of SG formation in response to H_2_O_2_ treatment has been a source of debate [37,38]. It should be noted that, in most previously published reports, H_2_O_2_ by itself triggers SG assembly only at concentrations above 1 mM, which may be due to the significant inhibition of translation initiation at such elevated concentrations of H_2_O_2_ [36]. Remarkably, we observed that 1mM H_2_O_2_-induced SG assembly in the cytoplasm is controlled by PARP1 (Figure 6B). Milder stress conditions, H_2_O_2_ at 300 µM, are sufficient to trigger a very efficient PARP1 activation in HeLa cells, but not SG formation (Figure 2B). Furthermore, when cells are pretreated by puromycin, which counteracts the elongation block and promotes polysome dissociation, H_2_O_2_ at 300 µM strongly induces the assembly of SGs (Figure 2B). In addition, SG assembly in puromycin/H_2_O_2_-treated cells is strictly dependent on PARP1 activity (Figure 2A,C,D). Thus, if translation initiation is inhibited by a high dose of H_2_O_2_ or translation elongation is blocked by puromycin, causing the polysome dissociation, PARP1 activation promotes cytoplasmic SG assembly (Figure 6B). 

### 4.3. PARP1 Activation Positively Regulates Pre-Formed Nuclear and Cytoplasmic mRNA-Rich Granules but Does Not Induce PAR Accumulation in SGs

Although the putative role of cytoplasmic PARPs in the formation of SGs has been already shown [9,10,11,12], the direct involvement of PARP1 in the building of SGs is not fully understood [22]. Here, we found that the presence and accumulation of H_2_O_2_-induced cytoplasmic, but more importantly, nuclear, SG-like structures directly depend on PARP1 activity. We also detected a co-localization of MAR/PAR with FUS or TDP-43 in cytoplasmic granules that were formed under H_2_O_2_-induced stress conditions in HeLa cell-overexpressed FUS (or TDP-43) (Figure 3C). This fact may be in agreement with the hypothesis that PAR is a scaffold for triggering the assembly of specific sets of proteins in condensates [14], as observed with purified FUS and PAR in vitro [15,30,67]. However, we failed to detect an increase in PAR enrichment in SGs, whether they were induced by arsenite or H_2_O_2_ treatment. In spite of the fact that the H_2_O_2_-induced SG formation is upregulated by PARP1 activity, our data do not provide additional support for a role of PAR as a scaffold promoting the assembly of mRNA-rich granules under this stress condition. Nevertheless, PARP1 activation could drive the translocation of FUS to the cytoplasm, and H_2_O_2_-induced cytoplasmic SGs are much more abundant (Figure 6C). 

We also analyzed PAR enrichment in mRNA-rich granules in the nucleus, formed after H_2_O_2_ treatment in combination with ActD, and found that H_2_O_2_ enhances the nuclear granules’ formation and their enrichment of mRNA, and their effect is weakened by the PARP1 inhibitor; nevertheless, we again failed to observe an enrichment of PAR in these nuclear granules (Figure 4). Thus, it is possible that condensates formed by mRNA or PAR do not mix or fuse with each other, which is important to investigate further. 

### 4.4. PARP1 Activation Promotes Assembly of Pre-Formed SGs and Prevents Their Dissociation 

Although the pretreatment of cells by H_2_O_2_ strongly inhibits SG formation after arsenite stress (Figure 1A), the dissociation of SGs pre-formed in the presence of arsenite is prevented in a PARP1-dependent manner (Figure 5A,B and Figure 6D). In the case of cytoplasmic pre-formed TDP-43- and FUS-rich granules, H_2_O_2_ exposure caused a significant increase in the number of FUS-rich granules in a PARP1-dependent manner (Figure 5C,D and Figure 6C). Both TDP-43 and FUS translocate from the nucleus to the cytoplasm during aging in neurons of ALS and FTLD patients [49,68] and form cytoplasmic inclusions that can participate directly in the progression of these diseases. Finding a link between PARP1 activation and the maintenance of TDP-43 and FUS condensates could be of interest since recent reports propose a critical role for poly(ADP-ribosyl)ation in association with neurodegeneration [20,26,28,29]. 

However, we cannot conclude whether preventing SG assembly would be beneficial or detrimental for the progression of neurodegenerative diseases [69]. Since the presence of TDP-43 or FUS in SGs may either promote the formation of protein aggregates, for instance, a local increase in TDP-43 and FUS in SGs [65], or inhibit their formation, we may consider that mRNA present in SGs behaves as TDP-43 and FUS chaperones to prevent a transition from reversible liquid-like condensates to irreversible protein aggregates [12,52,54]. 

## 5. Conclusions

The formation of H_2_O_2_-induced cytoplasmic and nuclear mRNA-rich granules directly correlates with PARP1 activity. In sum, three hypotheses can be proposed: (i) SG protein components are PARylated, including FUS [70], which may in turn promote RNA granule assembly; (ii) PARP1 activation may also activate signals via phosphorylation events [71,72] to force the assembly of mRNA-rich granules; (iii) as PAR is not found inside nuclear mRNA-rich granules after PARP1 activation (Figure 3, Figure 4 and Figure S3A), PAR may compete with mRNA for binding to RBPs [30,67]. In this latter model, mRNA-stabilizing proteins would rather bind PAR than mRNA. PAR would then promote the formation of pre-formed mRNA granules without being part of these granules itself. However, this model is more likely to take place in the nucleus than in the cytoplasm, owing to the net increase in PAR in the nucleus after PARP1 activation, but we cannot exclude that nuclear PAR may prevent the shuttling of nuclear mRNA-stabilizing proteins to the cytoplasm, as observed with HuR (Supplementary Figure S5A,B).

## Data Availability

Not applicable.

## Supplementary Materials

The following supporting information can be downloaded at: https://www.mdpi.com/article/10.3390/cells11233932/s1, Figure S1. Exposure of cells to H2O2, in contrast to arsenite, leads to a strong activation of PARP1, but does not cause significant phosphorylation of eIF2A; Figure S2. PARP1 activity contributes to stress assembly; Figure S3. PARP1 activity contributes to stress assembly if the polysomes were previously destabilized; Figure S4. PAR enrichment was not observed in SG formed in the H2O2- and arsenite-induced stress conditions; Figure S5. Western blot analysis of PARG expression; Figure S6. PARP1 activation may prevent the shuttling of HuR to the cytoplasm; Figure S7. Analysis of stress granule composition and assembly; Table S1 and Table S2.

## References

[1] Protter D.S.W., Parker R. (2016). Principles and Properties of Stress Granules. Trends Cell Biol..

[2] Aulas A., Fay M.M., Lyons S.M., Achorn C.A., Kedersha N., Anderson P., Ivanov P. (2017). Stress-Specific Differences in Assembly and Composition of Stress Granules and Related Foci. J. Cell Sci..

[3] Markmiller S., Soltanieh S., Server K.L., Mak R., Jin W., Fang M.Y., Luo E.C., Krach F., Yang D., Sen A. (2018). Context-Dependent and Disease-Specific Diversity in Protein Interactions within Stress Granules. Cell.

[4] Bounedjah O., Desforges B., Wu T.-D., Pioche-Durieu C., Marco S., Hamon L., Curmi P.A., Guerquin-Kern J.-L., Piétrement O., Pastré D. (2014). Free MRNA in Excess upon Polysome Dissociation Is a Scaffold for Protein Multimerization to Form Stress Granules. Nucleic Acids Res..

[5] van Treeck B., Protter D.S.W., Matheny T., Khong A., Link C.D., Parker R. (2018). RNA Self-Assembly Contributes to Stress Granule Formation and Defining the Stress Granule Transcriptome. Proc. Natl. Acad. Sci. USA.

[6] Guillén-Boixet J., Kopach A., Holehouse A.S., Wittmann S., Jahnel M., Schlüßler R., Kim K., Trussina I.R.E.A., Wang J., Mateju D. (2020). RNA-Induced Conformational Switching and Clustering of G3BP Drive Stress Granule Assembly by Condensation. Cell.

[7] Sanders D.W., Kedersha N., Lee D.S.W., Strom A.R., Drake V., Riback J.A., Bracha D., Eeftens J.M., Iwanicki A., Wang A. (2020). Competing Protein-RNA Interaction Networks Control Multiphase Intracellular Organization. Cell.

[8] Yang P., Mathieu C., Kolaitis R.M., Zhang P., Messing J., Yurtsever U., Yang Z., Wu J., Li Y., Pan Q. (2020). G3BP1 Is a Tunable Switch That Triggers Phase Separation to Assemble Stress Granules. Cell.

[9] Duan Y., Du A., Gu J., Duan G., Wang C., Gui X., Ma Z., Qian B., Deng X., Zhang K. (2019). PARylation Regulates Stress Granule Dynamics, Phase Separation, and Neurotoxicity of Disease-Related RNA-Binding Proteins. Cell Res..

[10] Jin X., Cao X., Liu S., Liu B. (2021). Functional Roles of Poly(ADP-Ribose) in Stress Granule Formation and Dynamics. Front. Cell Dev. Biol..

[11] Leung A.K.L., Vyas S., Rood J.E., Bhutkar A., Sharp P.A., Chang P. (2011). Poly(ADP-Ribose) Regulates Stress Responses and MicroRNA Activity in the Cytoplasm. Mol. Cell.

[12] McGurk L., Gomes E., Guo L., Mojsilovic-Petrovic J., Tran V., Kalb R.G., Shorter J., Bonini N.M. (2018). Poly(ADP-Ribose) Prevents Pathological Phase Separation of TDP-43 by Promoting Liquid Demixing and Stress Granule Localization. Mol. Cell.

[13] Lüscher B., Ahel I., Altmeyer M., Ashworth A., Bai P., Chang P., Cohen M., Corda D., Dantzer F., Daugherty M.D. (2021). ADP-ribosyltransferases, an Update on Function and Nomenclature. FEBS J..

[14] Leung A.K.L. (2020). Poly(ADP-Ribose): A Dynamic Trigger for Biomolecular Condensate Formation. Trends Cell Biol..

[15] Altmeyer M., Neelsen K.J., Teloni F., Pozdnyakova I., Pellegrino S., Grøfte M., Rask M.-B.D., Streicher W., Jungmichel S., Nielsen M.L. (2015). Liquid Demixing of Intrinsically Disordered Proteins Is Seeded by Poly(ADP-Ribose). Nat. Commun..

[16] Alemasova E.E., Pestryakov P.E., Sukhanova M.v., Kretov D.A., Moor N.A., Curmi P.A., Ovchinnikov L.P., Lavrik O.I. (2015). Poly(ADP-Ribosyl)Ation as a New Posttranslational Modification of YB-1. Biochimie.

[17] Isabelle M., Gagné J.-P., Gallouzi I.-E., Poirier G.G. (2012). Quantitative Proteomics and Dynamic Imaging Reveal That G3BP-Mediated Stress Granule Assembly Is Poly(ADP-Ribose)-Dependent Following Exposure to MNNG-Induced DNA Alkylation. J. Cell Sci..

[18] Chen K., Zhang J., Beeraka N.M., Tang C., Babayeva Y.V., Sinelnikov M.Y., Zhang X., Zhang J., Liu J., Reshetov I.V. (2022). Advances in the Prevention and Treatment of Obesity-Driven Effects in Breast Cancers. Front. Oncol..

[19] Chen K., Lu P., Beeraka N.M., Sukocheva O.A., Madhunapantula S.R.V., Liu J., Sinelnikov M.Y., Nikolenko V.N., Bulygin K.V., Mikhaleva L.M. (2022). Mitochondrial Mutations and Mitoepigenetics: Focus on Regulation of Oxidative Stress-Induced Responses in Breast Cancers. Semin. Cancer Biol..

[20] Walker C., El-Khamisy S.F. (2018). Perturbed Autophagy and DNA Repair Converge to Promote Neurodegeneration in Amyotrophic Lateral Sclerosis and Dementia. Brain.

[21] Bai P. (2015). Biology of Poly(ADP-Ribose) Polymerases: The Factotums of Cell Maintenance. Mol. Cell.

[22] Catara G., Grimaldi G., Schembri L., Spano D., Turacchio G., lo Monte M., Beccari A.R., Valente C., Corda D. (2017). PARP1-Produced Poly-ADP-Ribose Causes the PARP12 Translocation to Stress Granules and Impairment of Golgi Complex Functions. Sci. Rep..

[23] Ayyappan V., Wat R., Barber C., Vivelo C.A., Gauch K., Visanpattanasin P., Cook G., Sazeides C., Leung A.K.L. (2021). ADPriboDB 2.0: An Updated Database of ADP-Ribosylated Proteins. Nucleic Acids Res..

[24] Bonfiglio J.J., Fontana P., Zhang Q., Colby T., Gibbs-Seymour I., Atanassov I., Bartlett E., Zaja R., Ahel I., Matic I. (2017). Serine ADP-Ribosylation Depends on HPF1. Mol. Cell.

[25] Martello R., Leutert M., Jungmichel S., Bilan V., Larsen S.C., Young C., Hottiger M.O., Nielsen M.L. (2016). Proteome-Wide Identification of the Endogenous ADP-Ribosylome of Mammalian Cells and Tissue. Nat. Commun..

[26] McGurk L., Rifai O.M., Bonini N.M. (2019). Poly(ADP-Ribosylation) in Age-Related Neurological Disease. Trends Genet..

[27] Thapa K., Khan H., Sharma U., Grewal A.K., Singh T.G. (2021). Poly (ADP-Ribose) Polymerase-1 as a Promising Drug Target for Neurodegenerative Diseases. Life Sci..

[28] Liu C., Fang Y. (2019). New Insights of Poly(ADP-Ribosylation) in Neurodegenerative Diseases: A Focus on Protein Phase Separation and Pathologic Aggregation. Biochem. Pharm..

[29] Park H., Kam T.-I., Dawson T.M., Dawson V.L. (2020). Poly (ADP-Ribose) (PAR)-Dependent Cell Death in Neurodegenerative Diseases. International Review of Cell and Molecular Biology.

[30] Singatulina A.S., Hamon L., Sukhanova M.V., Desforges B., Joshi V., Bouhss A., Lavrik O.I., Pastré D. (2019). PARP-1 Activation Directs FUS to DNA Damage Sites to Form PARG-Reversible Compartments Enriched in Damaged DNA. Cell Rep..

[31] da Cruz S., Cleveland D.W. (2011). Understanding the Role of TDP-43 and FUS/TLS in ALS and Beyond. Curr. Opin. Neurobiol..

[32] Dobra I., Pankivskyi S., Samsonova A., Pastre D., Hamon L. (2018). Relation Between Stress Granules and Cytoplasmic Protein Aggregates Linked to Neurodegenerative Diseases. Curr. Neurol. Neurosci. Rep..

[33] Panda A.C., Martindale J.L., Gorospe M. (2017). Polysome Fractionation to Analyze MRNA Distribution Profiles. Bio. Protoc..

[34] Kedersha N., Chen S., Gilks N., Li W., Miller I.J., Stahl J., Anderson P. (2002). Evidence That Ternary Complex (EIF2-GTP-TRNAiMet)-Deficient Preinitiation Complexes Are Core Constituents of Mammalian Stress Granules. Mol. Biol. Cell.

[35] Erdélyi K., Bai P., Kovács I., Szabó É., Mocsár G., Kakuk A., Szabó C., Gergely P., Virág L. (2009). Dual Role of Poly(ADP-ribose) Glycohydrolase in the Regulation of Cell Death in Oxidatively Stressed A549 Cells. FASEB J..

[36] Emara M.M., Fujimura K., Sciaranghella D., Ivanova V., Ivanov P., Anderson P. (2012). Hydrogen Peroxide Induces Stress Granule Formation Independent of EIF2α Phosphorylation. Biochem. Biophys. Res. Commun..

[37] Pothof J., Verkaik N.S., Hoeijmakers J.H., van Gent D.C. (2009). Cell Cycle MicroRNA Responses and Stress Granule Formation Modulate the DNA Damage Response. Taylor Fr..

[38] Wolf A., Krause-Gruszczynska M., Birkenmeier O., Ostareck-Lederer A., Hüttelmaier S., Hatzfeld M. (2010). Plakophilin 1 Stimulates Translation by Promoting EIF4A1 Activity. J. Cell Biol..

[39] Brown J.A.L., Roberts T.L., Richards R., Woods R., Birrell G., Lim Y.C., Ohno S., Yamashita A., Abraham R.T., Gueven N. (2011). A Novel Role for HSMG-1 in Stress Granule Formation. Mol. Cell Biol..

[40] Arimoto-Matsuzaki K., Saito H., Takekawa M. (2016). TIA1 Oxidation Inhibits Stress Granule Assembly and Sensitizes Cells to Stress-Induced Apoptosis. Nat. Commun..

[41] Thedieck K., Holzwarth B., Prentzell M.T., Boehlke C., Kläsener K., Ruf S., Sonntag A.G., Maerz L., Grellscheid S.N., Kremmer E. (2013). XInhibition of MTORC1 by Astrin and Stress Granules Prevents Apoptosis in Cancer Cells. Cell.

[42] Arimoto-Matsuzaki K. (2008). Formation of Stress Granules Inhibits Apoptosis by Suppressing Stress-Responsive MAPK Pathways. Artic. Nat. Cell Biol..

[43] Takahashi M., Higuchi M., Matsuki H., Yoshita M., Ohsawa T., Oie M., Fujii M. (2013). Stress Granules Inhibit Apoptosis by Reducing Reactive Oxygen Species Production. Mol. Cell Biol..

[44] Thorsell A.-G., Ekblad T., Karlberg T., Löw M., Pinto A.F., Trésaugues L., Moche M., Cohen M.S., Schüler H. (2017). Structural Basis for Potency and Promiscuity in Poly(ADP-Ribose) Polymerase (PARP) and Tankyrase Inhibitors. J. Med. Chem..

[45] Sanchez M., Lin Y., Yang C.-C., McQuary P., Rosa Campos A., Aza Blanc P., Wolf D.A. (2019). Cross Talk between EIF2α and EEF2 Phosphorylation Pathways Optimizes Translational Arrest in Response to Oxidative Stress. iScience.

[46] Kedersha N., Cho M.R., Li W., Yacono P.W., Chen S., Gilks N., Golan D.E., Anderson P. (2000). Dynamic Shuttling of TIA-1 Accompanies the Recruitment of MRNA to Mammalian Stress Granules. J. Cell Biol..

[47] Beneke S., Meyer K., Holtz A., Hüttner K., Bürkle A. (2012). Chromatin Composition Is Changed by Poly(ADP-Ribosyl)Ation during Chromatin Immunoprecipitation. PLoS ONE.

[48] Slade D., Dunstan M.S., Barkauskaite E., Weston R., Lafite P., Dixon N., Ahel M., Leys D., Ahel I. (2011). The Structure and Catalytic Mechanism of a Poly(ADP-Ribose) Glycohydrolase. Nature.

[49] Naumann M., Pal A., Goswami A., Lojewski X., Japtok J., Vehlow A., Naujock M., Günther R., Jin M., Stanslowsky N. (2018). Impaired DNA Damage Response Signaling by FUS-NLS Mutations Leads to Neurodegeneration and FUS Aggregate Formation. Nat. Commun..

[50] Bosco D.A., Lemay N., Ko H.K., Zhou H., Burke C., Kwiatkowski T.J., Sapp P., McKenna-Yasek D., Brown R.H., Hayward L.J. (2010). Mutant FUS Proteins That Cause Amyotrophic Lateral Sclerosis Incorporate into Stress Granules. Hum. Mol. Genet..

[51] Colombrita C., Zennaro E., Fallini C., Weber M., Sommacal A., Buratti E., Silani V., Ratti A. (2009). TDP-43 Is Recruited to Stress Granules in Conditions of Oxidative Insult. J. Neurochem..

[52] Fang M.Y., Markmiller S., Vu A.Q., Javaherian A., Dowdle W.E., Jolivet P., Bushway P.J., Castello N.A., Baral A., Chan M.Y. (2019). Small-Molecule Modulation of TDP-43 Recruitment to Stress Granules Prevents Persistent TDP-43 Accumulation in ALS/FTD. Neuron.

[53] Gal J., Zhang J., Kwinter D.M., Zhai J., Jia H., Jia J., Zhu H. (2011). Nuclear Localization Sequence of FUS and Induction of Stress Granules by ALS Mutants. Neurobiol. Aging.

[54] Rengifo-Gonzalez J.C., el Hage K., Clément M.-J., Steiner E., Joshi V., Craveur P., Durand D., Pastré D., Bouhss A. (2021). The Cooperative Binding of TDP-43 to GU-Rich RNA Repeats Antagonizes TDP-43 Aggregation. Elife.

[55] Wheeler E.C., Vu A.Q., Einstein J.M., DiSalvo M., Ahmed N., van Nostrand E.L., Shishkin A.A., Jin W., Allbritton N.L., Yeo G.W. (2020). Pooled CRISPR Screens with Imaging on Microraft Arrays Reveals Stress Granule-Regulatory Factors. Nat. Methods.

[56] Youn J.-Y., Dunham W.H., Hong S.J., Knight J.D.R., Bashkurov M., Chen G.I., Bagci H., Rathod B., MacLeod G., Eng S.W.M. (2018). High-Density Proximity Mapping Reveals the Subcellular Organization of MRNA-Associated Granules and Bodies. Mol. Cell.

[57] Bley N., Lederer M., Pfalz B., Reinke C., Fuchs T., Glaß M., Möller B., Hüttelmaier S. (2015). Stress Granules Are Dispensable for MRNA Stabilization during Cellular Stress. Nucleic Acids Res..

[58] Páhi Z.G., Borsos B.N., Pantazi V., Ujfaludi Z., Pankotai T. (2020). PARylation During Transcription: Insights into the Fine-Tuning Mechanism and Regulation. Cancers.

[59] Child J.R., Chen Q., Reid D.W., Jagannathan S., Nicchitta C.V. (2021). Recruitment of Endoplasmic Reticulum-Targeted and Cytosolic MRNAs into Membrane-Associated Stress Granules. RNA.

[60] Wheeler J.R., Matheny T., Jain S., Abrisch R., Parker R. (2016). Distinct Stages in Stress Granule Assembly and Disassembly. Elife.

[61] O’Sullivan J., Tedim Ferreira M., Gagné J.-P., Sharma A.K., Hendzel M.J., Masson J.-Y., Poirier G.G. (2019). Emerging Roles of Eraser Enzymes in the Dynamic Control of Protein ADP-Ribosylation. Nat. Commun..

[62] Lagueux J., Shah G.M., Menard L., Thomassin H., Duchaine C., Hengartner C., Poirier G.G. (1994). Poly(ADP-Ribose) Catabolism in Mammalian Cells. Mol. Cell Biochem..

[63] Cao X., Jin X., Liu B. (2020). The Involvement of Stress Granules in Aging and Aging-Associated Diseases. Aging Cell.

[64] Chen L., Liu B. (2017). Relationships between Stress Granules, Oxidative Stress, and Neurodegenerative Diseases. Oxid. Med. Cell Longev..

[65] Li Y.R., King O.D., Shorter J., Gitler A.D. (2013). Stress Granules as Crucibles of ALS Pathogenesis. J. Cell Biol..

[66] Wolozin B., Ivanov P. (2019). Stress Granules and Neurodegeneration. Nat. Rev. Neurosci..

[67] Rhine K., Dasovich M., Yoniles J., Badiee M., Skanchy S., Ganser L.R., Ge Y., Fare C.M., Shorter J., Leung A.K.L. (2022). Poly(ADP-Ribose) Drives Condensation of FUS via a Transient Interaction. Mol. Cell.

[68] Ederle H., Dormann D. (2017). TDP-43 and FUS En Route from the Nucleus to the Cytoplasm. FEBS Lett..

[69] Gasset-Rosa F., Lu S., Yu H., Chen C., Melamed Z., Guo L., Shorter J., da Cruz S., Cleveland D.W. (2019). Cytoplasmic TDP-43 De-Mixing Independent of Stress Granules Drives Inhibition of Nuclear Import, Loss of Nuclear TDP-43, and Cell Death. Neuron.

[70] Jungmichel S., Rosenthal F., Altmeyer M., Lukas J., Hottiger M.O., Nielsen M.L. (2013). Proteome-Wide Identification of Poly(ADP-Ribosyl)Ation Targets in Different Genotoxic Stress Responses. Mol. Cell.

[71] Éthier C., Tardif M., Arul L., Poirier G.G. (2012). PARP-1 Modulation of MTOR Signaling in Response to a DNA Alkylating Agent. PLoS ONE.

[72] Cohen-Armon M. (2007). PARP-1 Activation in the ERK Signaling Pathway. Trends Pharmacol. Sci..

