# Supplementary material for: PARP1 Activation Controls Stress Granule Assembly after Oxidative Stress and DNA Damage
Source: Cells. 2022 Dec 5;11(23):3932. doi: 10.3390/cells11233932 (PMC9740212; doi:10.3390/cells11233932)
Supplement: Supplementary file 1 [file cells-11-03932-s001.zip › cells-2029706-supplementary.pdf]

SUPPLEMENTARY MATERIALS

Supplementary Figures

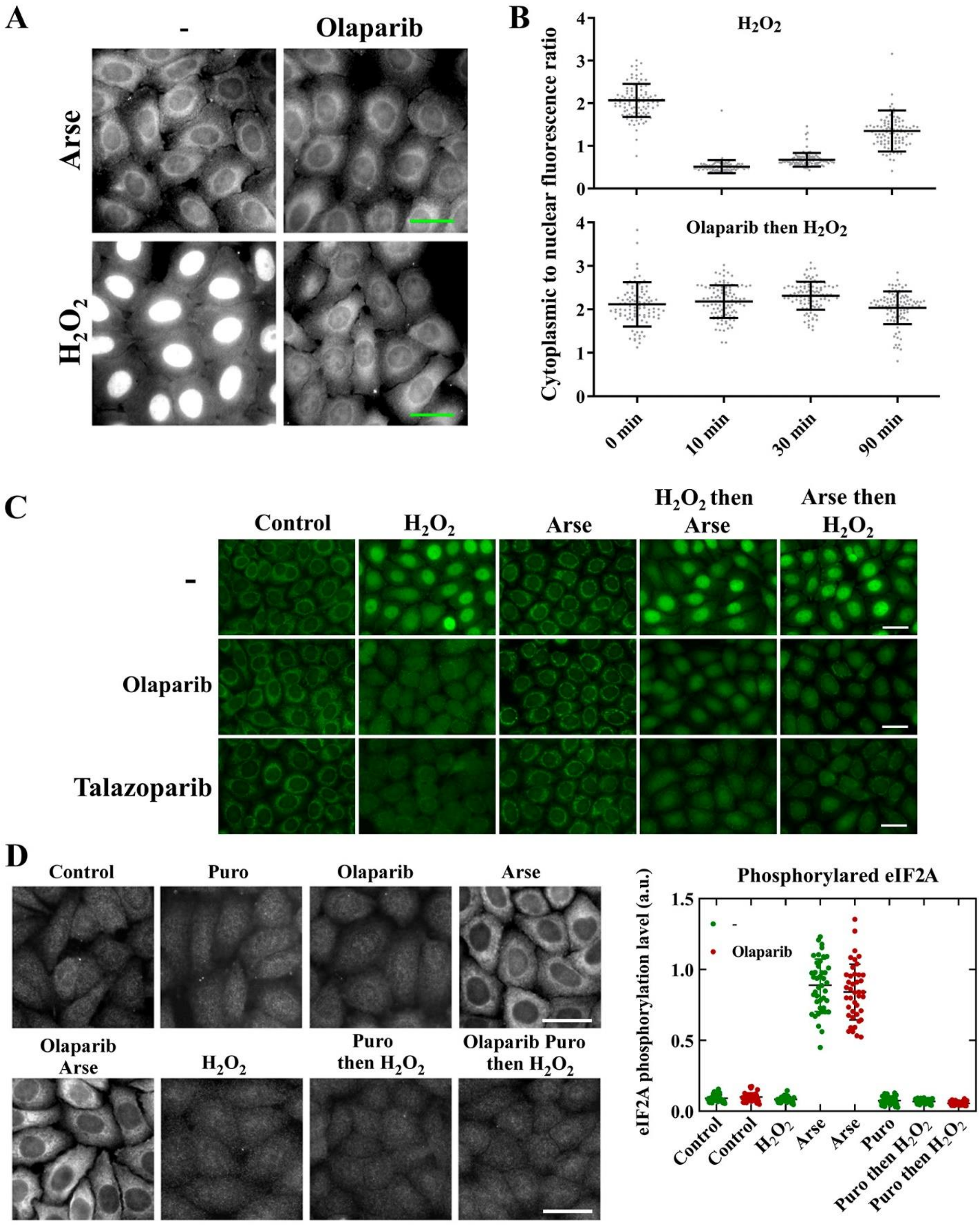

Figure S1. Exposure of cells to H_2O_2, in contrast to arsenite, leads to a strong activation of PARP1, but does not cause significant phosphorylation of eIF2A. (A) In contrast to arsenite, cell treatment with H_2O_2 triggers a massive synthesis of PAR in the nucleus. HeLa cells were exposed to either 300 µM H_2O_2 or 300µM arsenite for 60 min. Cells were pre-treated with olaparib (10 µM) for 30 min where indicated. Immunofluorescence staining: Anti-PAR antibody. Scale bar: 10 µm. **(B)** The relative distribution of PAR between the cytoplasm and nucleus of HeLa cells was measured at different times after H_2O_2 treatment (300 µM) with or without olaparib (10 µM) pre-treatment. The integrated fluorescence intensities were measured at the single-cell level by using cell profiler software and secondary antibody fluorescence. Horizontal bars indicate mean; n =100. **(C)** Hela cells were exposed to indicated treatments (300 µM H_2O_2 for 30 min; 300 µM arsenite for 60 min). DMSO (control), to inhibit PARP1 10 µM olaparib or 1 µM talazoparib were added 1 h prior to indicated treatment. Scale bar: 10 µm. **(D)** Figure S1D. eIF2A is strongly phosphorylated in cells treated with arsenite, but H2O2 weakly induces phosphorylation of eIF2A. Immunofluorescence images of HeLa treated with either arsenite (300 µM) for 60 min, H2O2 (300 µM) for 90 min, olaparib (10 µM) for 15 min, puromycin (2.5 µg/mL) for 30 min. Staining: anti-phosphorylated eIF2α (serine 51). Left panel: immunofluorescence analysis of the single cell level of eIF2α phosphorylation (S51) for indicated conditions. Cells were treated with either arsenite, H_2O_2, puromycin, olaparib. Staining: Anti-phosphorylated eIF2α (serine 51). We measured the integrated intensity at the single cell level. Horizontal bar: mean, n=48. Note the phosphorylation of eIF2α after arsenite but not H_2O_2 treatment.

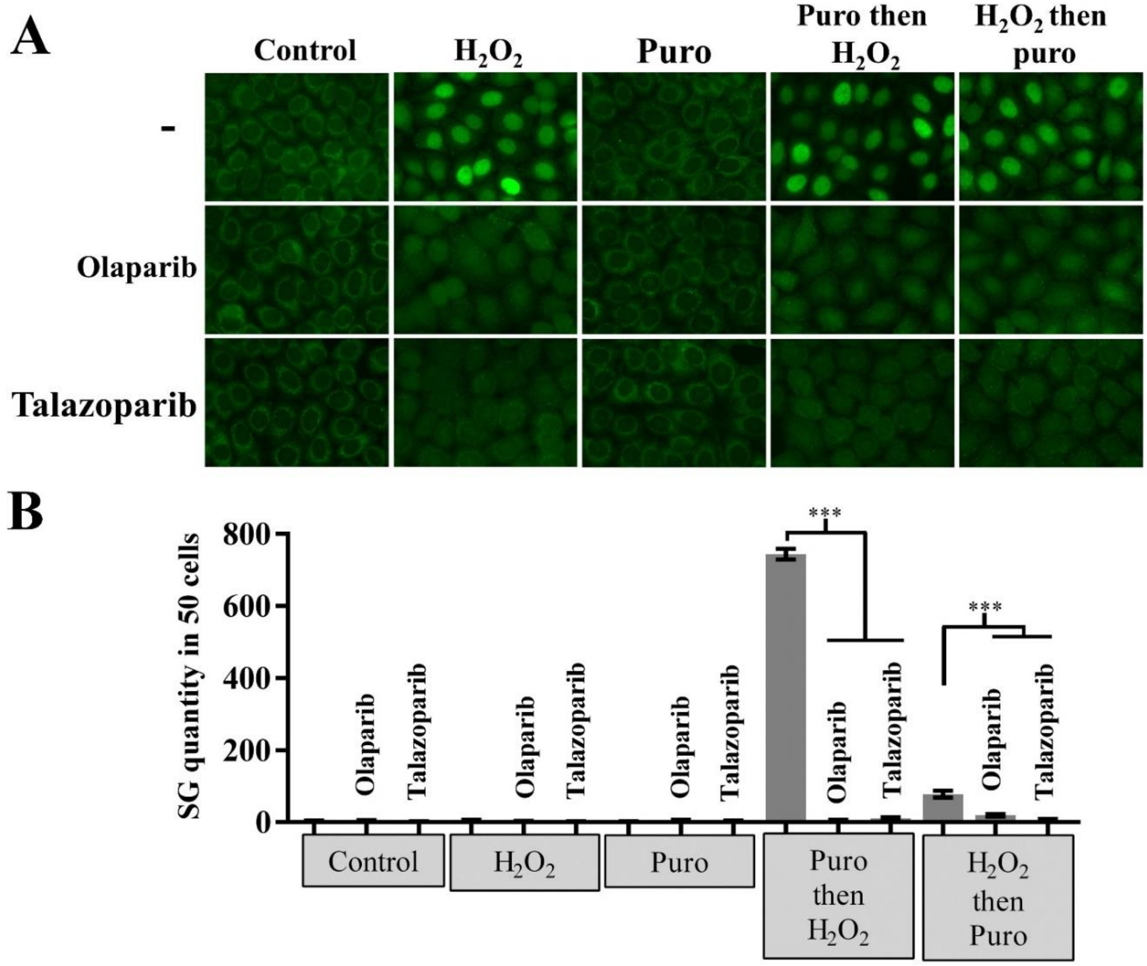

Figure S2. PARP1 activity contributes to stress assembly. (A) HeLa cells were exposed to indicated treatments (300 µM H_2O_2 for 30 min; 200 µM puromycin 60 min). DMSO (control), 10 µM olaparib or 1 µM talazoparib were added to 1h prior to indicated treatments to inhibit PARP1. Anti-PAR antibody. Scale bar: 10 µm. **(B)** PARP1 activity promotes stress granule assembly after polysome destabilization by puromycin. HeLa cells were exposed to indicated treatments (300 µM hydrogen peroxide for 30 min; 200 µM puromycin for 60 min). DMSO was used as a control, PARPs inhibitors, 10 µM olaparib or 1 µM talazoparib, were added to cells 1 h prior to indicated treatments. The number of stress granules for 50 cells were calculated for the indicated conditions. Stress granules were detected automatically by the presence of poly (A) mRNA (in situ hybridization) from cell images by using the Cell Profiler software. ***, $p < 0.001$; t test.

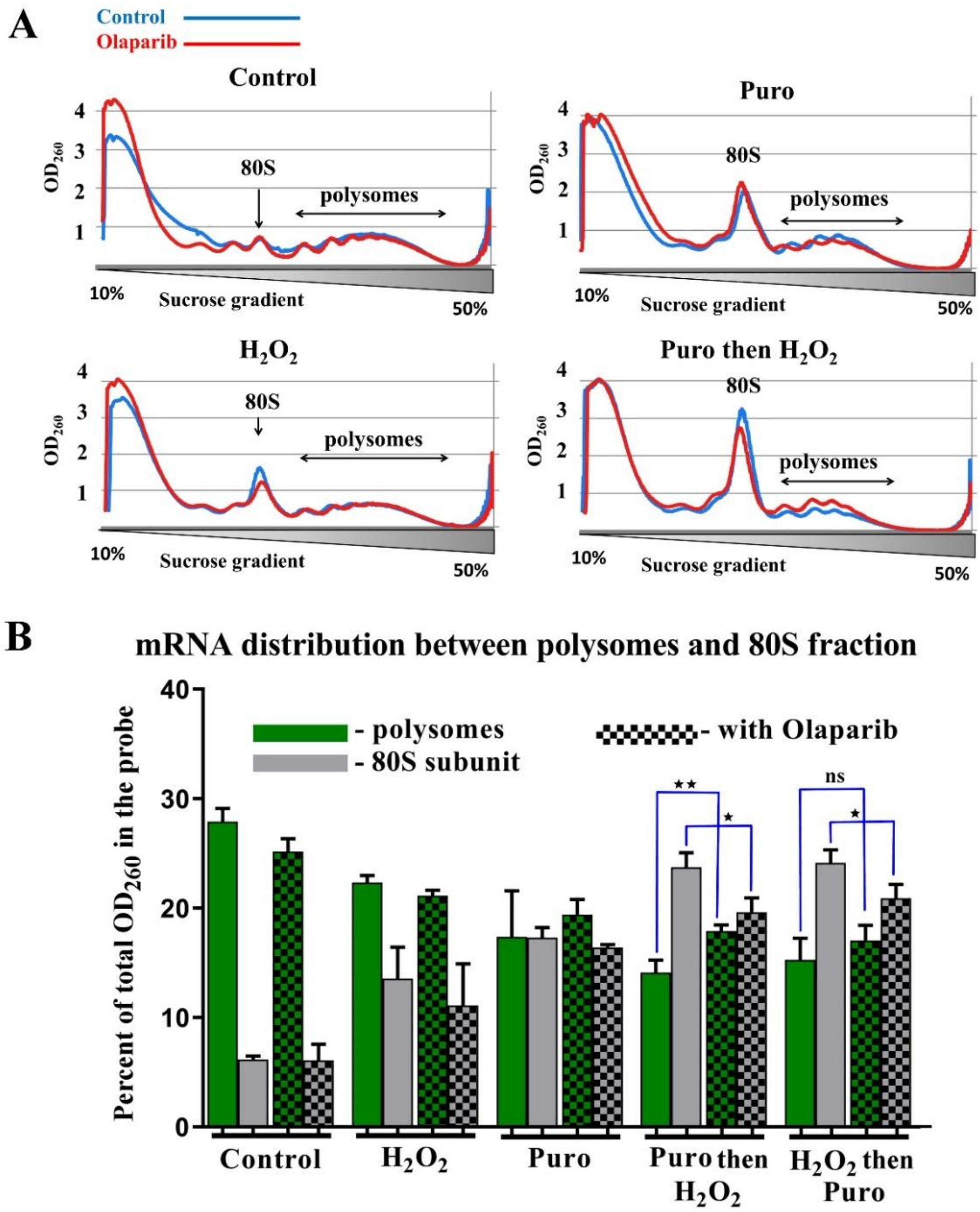

Figure S3. PARP1 activity contributes to stress assembly if the polysomes were previously destabilized. (A) Polysomal profiles showing influence of PARP1 activity on polysome dissociation when cells pretreated with puromycin. HeLa cells were exposed to indicated treatments (300 μM H_2O_2 for 30 min; 200 μM puromycin for 60 min). To inhibit PARP1, 10 μM olaparib was added to the cells 1 h prior to indicated treatments. Cytoplasmic lysates of HeLa cells exposed to indicated conditions were fractionated by sucrose gradient centrifugation. Polysome profiles are recorded at 260 nm and gradients were fractioned, fractions corresponding to polysomes and 80S ribosomes are indicated. **(B)** Analysis of mRNA distribution between polysomes and 80S fraction of sucrose gradient profiles (from (A) and with indicated conditions as in (A), normalized to total absorbance of the sample at the wavelength 260 nm.

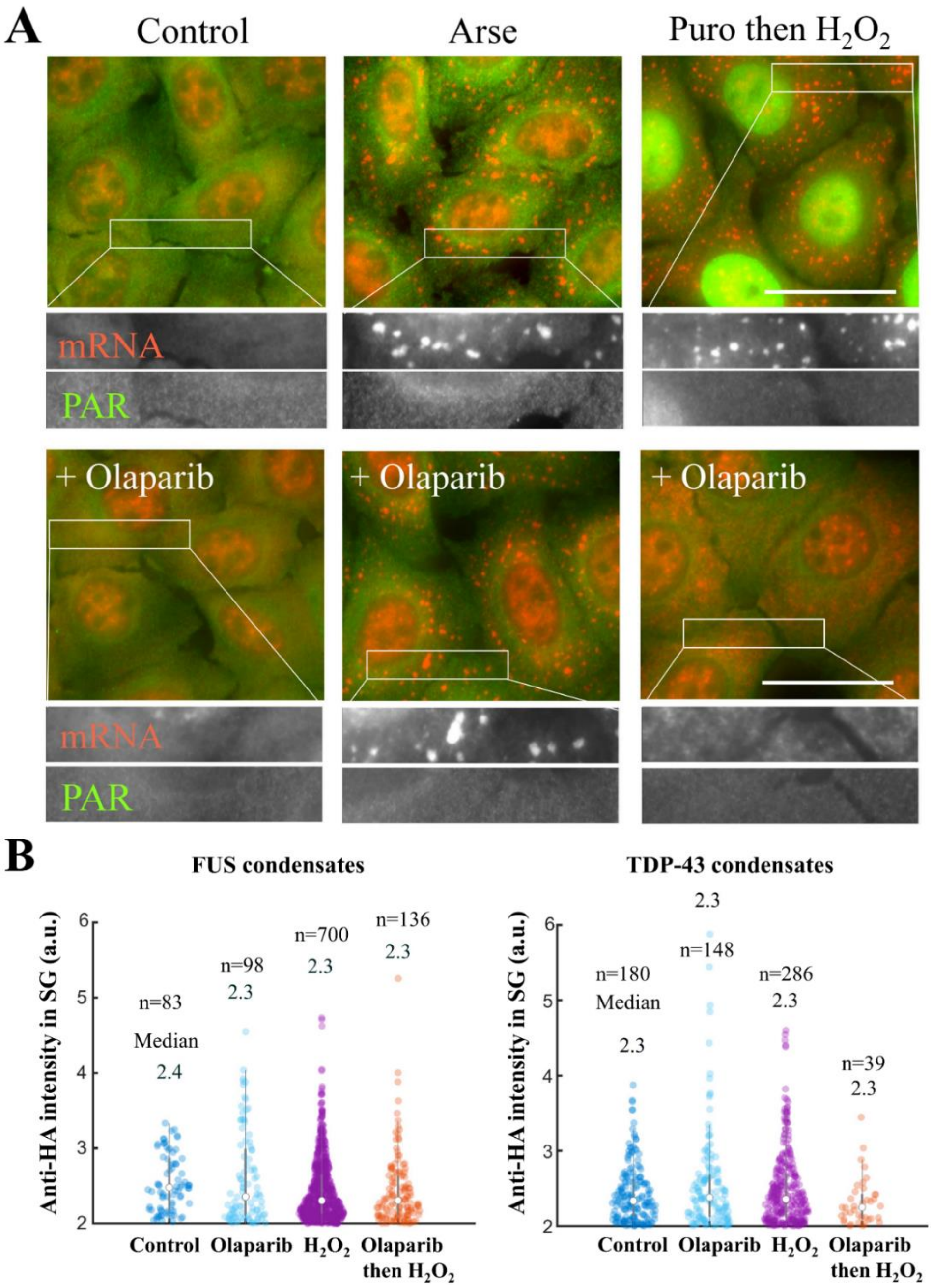

Figure S4. PAR enrichment was not observed in SG formed in the H_2O_2- and arsenite-induced stress conditions. (A) Before H_2O_2 treatment, cells were pre-treated with olaparib (10 μM) and puromycin (200 μM) for 30 min. Arsenite or H_2O_2 treatment: 300 μM for 60 min. Immunofluorescence staining: anti-PAR (green) antibodies; mRNA (red) - cy3-labeled poly(T)-probe. Scale bar: 10 μm. **(B)** The number of condensates and intensity of HA-tagged FUS or TDP-43 inside condensates after hydrogen peroxide treatment (300 μM for 1 h). "n" is the number of stress granules (condensates) detected for each condition. The level of HA-tagged protein in the condensates remains similar in all conditions.

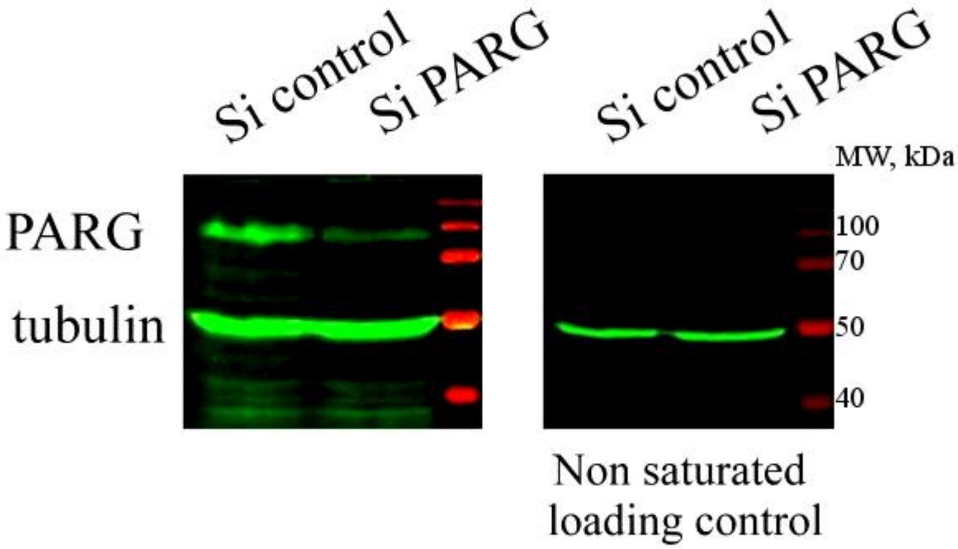

Figure S5. Western blot analysis of PARG expression. Western blot analysis of PARG expression in HeLa cell extracts treated with SiRNA control or SiRNA against PARG (SiPARG), measured 72 hours post transfection. Antibodies: anti-Alpha-tubulin (loading control) and anti-PARG. Cells were lysed and lysates were centrifuged at 14,000 × g for 15 min at 4 °C, then supernatants were collected. Proteins (120 μg) of cell extracts were separated on 12% SDS-PAGE gels and transferred onto a PVDF membrane. The membranes were blocked in 5% (w/v) non-fat dried milk and incubated for 1 h at room temperature with primary antibodies. Bound antibodies were detected and quantified using anti-mouse-IRDye 680RD secondary antibodies with an Odyssey imaging system.

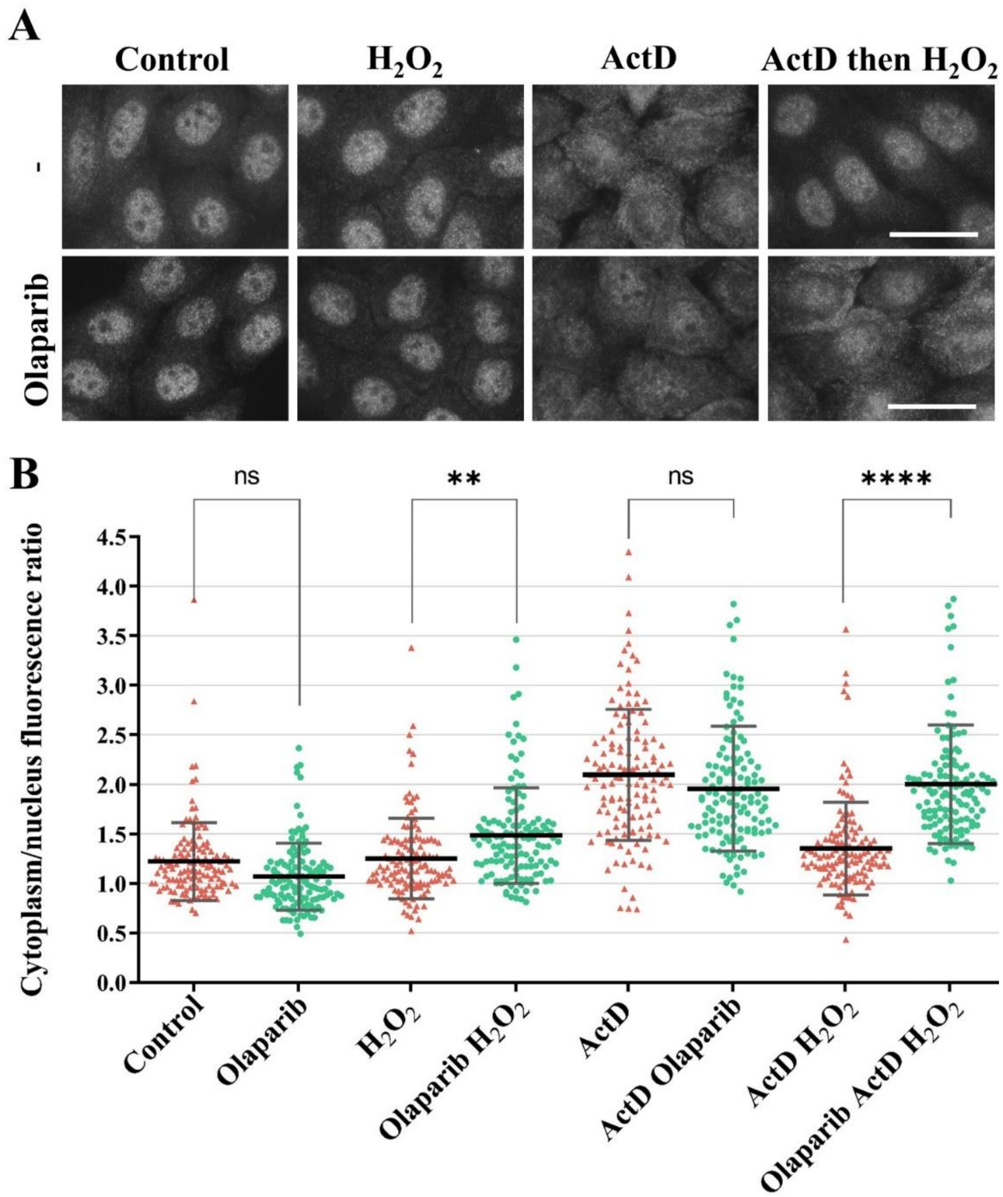

Figure S6. PARP1 activation may prevent the shuttling of HuR to the cytoplasm. (A) Spatial distribution of HuR in HeLa cells under indicated treatments (300 µM hydrogen peroxide for 1 h, DMSO (control), 4 µM actinomycin D for 1 h, 10 µM olaparib for 30min before indicated treatments). Scale bar: 10 µm. **(B)** Analysis of the nucleocytoplasmic shuttling of HuR. $p < 0.05$, ** paired t-test, n=130. H_2O_2 treatment prevents the translocation of HuR in response to ActD in HeLa cells in a PAR-dependent manner.

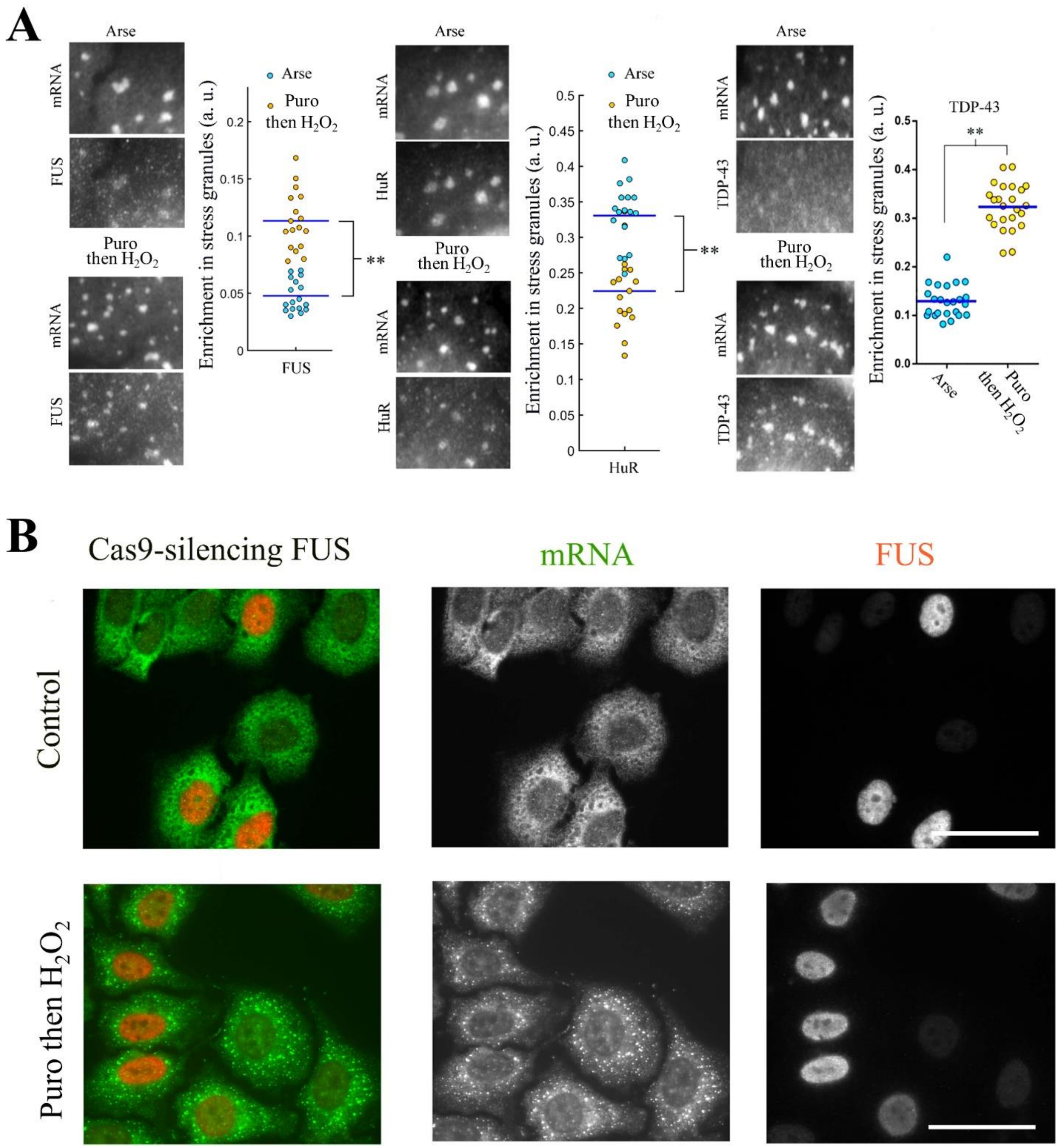

Figure S7. Analysis of stress granule composition and assembly. (**A**) The relative enrichment of FUS, HuR and TDP-43 in stress granules after H_2O_2/puromycin treatment. Immunofluorescence images of HeLa cells showing the distribution of FUS, HuR, TDP-43 (antibody) and mRNA (in situ hybridization) after exposure to either arsenite and H_2O_2/puromycin for 90 min. Ratio of protein to mRNA fluorescence integrated intensity in stress granules were measured under indicated conditions. $p < 0.05$, **; paired t-test, n=24. In contrast to HUR, the enrichment of FUS and TDP-43 is more pronounced in H_2O_2/puromycin- than arsenite-treated cells. Cell picture size: 2.3x2 µm. **(B)** Hela cells non-expressing FUS still form stress

granules after H_2O_2/puromycin treatment. The expression of FUS was shut down in HeLa cells using the CRISPR-Cas9 technology. HeLa cells that are not expressing FUS display a dark nucleus in contrast with the red nucleus of cells in which Cas9 was not expressed. mRNA (green). Anti-FUS (red). Scale bar: 10 µm. In brief, guide RNAs targeting human DNA encoding for FUS were designed using online resources (http://crispr.mit.edu). Corresponding oligonucleotides were then synthesized and subcloned into a vector (pX330, addgene plasmid #42230 or pX458, addgene plasmid #48138) containing the flanking gRNA sequences and a codon-optimized Cas9 cassette. Insert oligonucleotides were: FUS-F: cac cgc tga tta ctt caa gca gat; FUS-B: aaa cat ctg ctt gaa gta atc agc.5 days after transfection, the efficiency of FUS knockout in HeLa cells was probed by using immunofluorescence microscopy with an anti-FUS antibody. We noticed the presence of HeLa cells that were no longer expressing FUS (about 30%).

Supplementary Tables

Table S1

**Target**	**Primary antibodies**	**Supplier**	**References**
FUS/TLS	polyclonal anti-FUS, rabbit	Novus Biologicals	NB100-565
HuR	polyclonal anti-HuR / ELAVL1, mouse	Abcam	ab54987
TDP-43	anti-TDP-43/TARDBP, mouse	Santa Cruz Biotechnology	sc-376532
HA-Tag	Anti-HA-Tag (F-7)	Santa Cruz Biotechnology	sc-7392
Phospho-eIF2α	monoclonal anti-Phospho-eIF2α, rabbit	Cell Signaling Technology	3597L
PARG	monoclonal anti-PARG, mouse	Santa Cruz Biotechnology	sc-398563
Tubulin	monoclonal anti-Alpha-Tubulin, mouse	Abcam	ab7291

Table S2

**Materials and reagents**	**Supplier**	**References**
Goat anti-Rabbit IgG (H+L) Cross-Adsorbed Secondary Antibody, Alexa Fluor 594	Thermo Fisher Scientific	11012
Goat anti-Mouse IgG1 Cross-Adsorbed Secondary Antibody, Alexa Fluor 488	Thermo Fisher Scientific	21121
Goat anti-Mouse IgG, 680IRDye 680RD	LI-COR	926-68070
Olaparib (AZD2281, Ku-0059436)	Apexbio Technology	A4154

Talazoparib (1207456-01-6, BMN-673)	MedChemexpress	HY-108413
Oligo d(T)40 Cy3-conjugated oligonucleotides	Sigma	
Puromycin dihydrochloride from Streptomyces alboniger	Sigma	P8833
DMEM, penicillin-streptomycin, FBS	Gibco Life Technologies	N/a
